# Sensory and metabolite migration from tilapia skin to soup during the boiling process: fast and then slow

**DOI:** 10.1038/s41538-022-00168-w

**Published:** 2022-11-11

**Authors:** Jiahui Chen, Yinghua Nie, Jiamin Xu, Shudan Huang, Jie Sheng, Xichang Wang, Jian Zhong

**Affiliations:** 1 Xinhua Hospital, Shanghai Institute for Pediatric Research, Shanghai Key Laboratory of Pediatric Gastroenterology and Nutrition, Shanghai Jiao Tong University School of Medicine, Shanghai, 200092 China; 2 National R&D Branch Center for Freshwater Aquatic Products Processing Technology (Shanghai), Integrated Scientific Research Base on Comprehensive Utilization Technology for By-Products of Aquatic Product Processing, Ministry of Agriculture and Rural Affairs of the People’s Republic of China, Shanghai Engineering Research Center of Aquatic-Product Processing and Preservation, College of Food Science & Technology, Shanghai Ocean University, Shanghai, 201306 China

**Keywords:** Metabolomics, Mass spectrometry, Metabolomics, Metabolomics

## Abstract

This study mainly studied sensory and metabolite migration from the skin to the soup in the boiling process of tilapia skin using content analysis, electronic nose technique, electronic tongue technique, and metabolomics technique based on ultra-high performance liquid chromatography-mass spectrometry/mass spectrometry and gas chromatography-time-of-flight-mass spectrometry. The content changes, flavor changes, taste changes, metabolite numbers and differential metabolite numbers for both tilapia skin and soup mainly occurred in the initial 30 min. Moreover, the initial 10 min was the key period for the metabolite changes in the boiling process. Further, the differential metabolites in these three periods (0–10, 10–30, and 30–60 min) were identified to show the metabolites migration process. Six (adenine, gingerol, terephthalic acid, vanillin, pentanenitrile, and 2-pyrrolidinonede) and seven (butyramide, lysope(0:0/20:4(5z,8z,11z,14z)), lysope(22:6(4z,7z,10z,13z,16z,19z)/0:0), linoleic acid, N-acetylneuraminic acid, L-threose, and benzoin) chemicals were screened out in the differential metabolites of tilapia skin and soup, respectively, with Variable Importance in the Projection of >1 and *p* value of <0.05. This work would be beneficial to understand the sensory and metabolite migration in the preparation process of fish soup and provided a metabolomic analysis route to analyze metabolites migration in food.

## Introduction

Animal skin soup, a traditional Chinese food, has been generally thought as a good food therapy choice for beauty care, skin care, health care, and disease care in China for a long time. It can be dated back to the East Han dynasty, in which the medical saint Zhang Zhongjing (about 150~154 AD to 215~219 AD) developed pig skin soup with honey and fried rice for the treatment of dry sore throat, hoarse voice, and red or less moss tongue. This soup recipe was written in his books named “Shanghan Lun (Treatise on Febrile Diseases)” and “Jinkui Yaolue (Synopsis of the Golden Chamber)”^1^. So far, many skin soups have been developed from pig skin, cow skin, chicken skin, fish skin, etc.

Tilapia skin is a common by-product in tilapia processing factories^2^. Currently, tilapia skin is generally used to fabricate low-value feeds. It has been explored as xenograft for the treatment of burned skin of adults and children due to its excellent advantages such as noninfectious microbiota, similar morphology to human skin, and high tensile extension at break^3,4^. Because of high collagen content, tilapia skin has also been used to extract collagens^5,6^, gelatins^7,8^, and collagen peptides^9,10^ for potential food and medical applications. These methods provided some potential routes to value-addedly utilize tilapia skin. However, to the best of our knowledge, tilapia skin soup has not been sold in the market. Moreover, the sensory and metabolite migration in the boiling process of tilapia skin has not been studied to analyze their potential aroma and nutritional values.

Mass spectrometry-based untargeted metabolomics is an emerging technology to identify metabolites in biological systems and to analyze metabolite changes as a result of genetic, dietary, or environmental factors^11^. In the field of food science, it has been applied for food component analysis, food safety assessment, food consumption monitoring, and physiological monitoring of diet and nutrition studies^12,13^. Due to its excellent ability on food component analysis, it has been used to analyze the metabolites in the raw Pu’er tea^14^, Fu brick tea^15^, pinot noir wine^16^, four sea cucumber varieties (green, white, purple and spiny)^17^, thermal processed tilapia muscle^18^, and pufferfish soup^19^. These papers suggested mass spectrometry-based untargeted metabolomics was a powerful food component analysis tool for aroma profile comparison, sensory characteristics formation analysis, food origin prediction, metabolite profile comparison, and food processing effect analysis.

The purpose of this paper was to analyze the sensory and metabolite migration in the boiling process of tilapia skin. First, the content changes of tilapia skin and soup were analyzed. Second, the sensory changes of tilapia skin and soup were studied by electronic-nose (E-nose) and electronic-tongue (E-tongue) techniques. Final, metabolite migration from tilapia skin to soup were investigated by metabolomics statistical analyses, differential metabolite analyses, and metabolic pathway analyses. All results showed the sensory and metabolite migration was fast and then slow in the boiling process of tilapia skin.

## Results and discussion

### Content changes of tilapia skin and soup in the boiling process

**Fig. 1:**
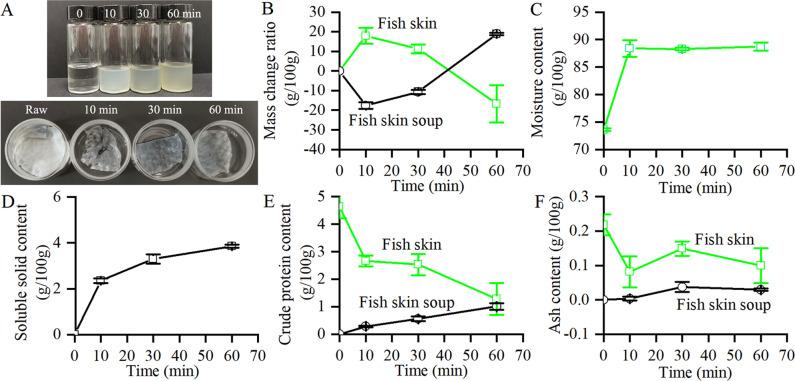
Content analysis of tilapia skin and soup in the boiling process. **A** Digital images of tilapia skin in 30 mL weighing bottle with a height of 3 cm and an inner diameter of 5 cm (the bottom image) and tilapia skin soup in 30 mL glass vial with a height of 7 cm and an inner diameter of 2.5 cm (the upper image). **B** Mass loss ratios of tilapia skin and tilapia skin soup. **C** Moisture content of tilapia skin. **D** Soluble solid content in tilapia skin soup. **E** Crude protein content. **F** Ash content in tilapia skin and soup. The error bars represent standard deviation (*n* = 3).

During the boiling process (Fig. 1A), tilapia skin soup became white (10 min) and then yellow (30 min and 60 min). It suggested that substance migration from tilapia skin to soup mainly occurred at 0–30 min. Tilapia skins still showed obvious skin shapes after the boiling process. The mass of fish skin increased at 0–10 min and then decreased at 10–60 min (Fig. 1B), whereas the mass of fish skin soup decreased at 0–10 min and then increased at 10–60 min (Fig. 1B). Moisture content of fish skin increased at 0–10 min and then showed no obvious changes at 10–60 min (Fig. 1C). Soluble solid content of fish skin soup increased with time (Fig. 1D). Crude protein content of fish skin decreased, whereas crude protein content of fish skin soup increased (Fig. 1E). It was consistent with that collagen and gelatin could be extracted into water after boiling tilapia skin^5,7^. Ash content of fish skin decreased and then fluctuated, whereas ash content of fish skin soup increased (Fig. 1F), which suggested that fish skin was adsorbed by water at the first 10 min and then was partially solubilized into fish skin soup (mainly 0–30 min). All these results suggested the substance migration were: 0–10 min period > 10–30 min period > 30–60 min period.

### Sensory changes of tilapia skin and soup in the boiling process

**Fig. 2:**
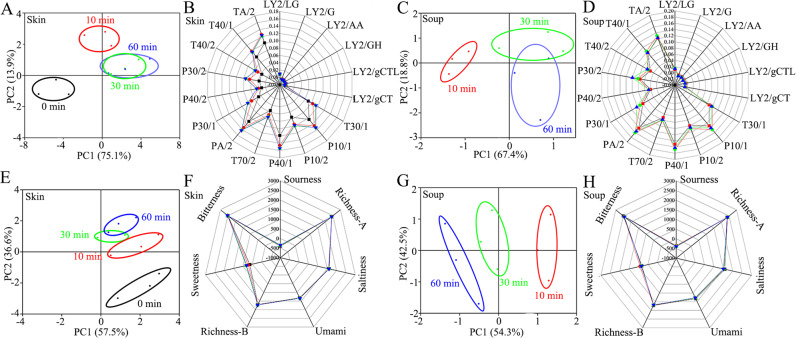
E-nose and E-tongue analyses of tilapia skin and soup in the boiling process. **A**, **B** E-nose PCA plot and radar graph of tilapia skin. **C**, **D**: E-nose PCA plot and radar graph of tilapia skin soup. **E**, **F** E-tongue PCA plot and radar graph of tilapia skin. **G**, **H** E-tongue PCA plot and radar graph of tilapia skin soup.

E-nose technique was applied to detect the changes of odors during the boiling process (Fig. 2A–D, and Supplementary Tables 1–3). Principal component analysis (PCA) plots were used to show the volatile components of tilapia skin and skin soup samples at different time points, as shown in Figs. 2A and C. Considering the tilapia skin soup at 0 min is actually ultrapure water, the ultrapure water was not detected in this work for the sensory and metabolite migration analyses. The first two components (PC1 was 75.1% and PC2 was 13.9%) explained that 89.0% of the total variance for tilapia skin (Fig. 2A). The first two components (PC1 was 67.4% and PC2 was 18.8%) explained that 86.2% of the total variance for tilapia skin soup (Fig. 2C). The measured data of each group were in a small oval shape, indicating that the E-nose data had good stability and repeatability. With the prolongation of the boiling time, the volatile components of tilapia skin changed. There was no overlapping area between the odor response values of tilapia skin at 10 and 30 min, indicating that the E-nose data could distinguish the samples well. The corresponding areas of 30 min and 60 min tilapia skins completely overlapped, which might indicate that the flavor of tilapia skin changed slower after 30 min. The flavor response values of the raw tilapia skin and the tilapia skin after boiling for 10 min were far apart, but the flavor response values of the tilapia skin after boiling for 10 min and 30 min were relatively close. The flavor of cooked tilapia skin was very different from that of raw tilapia skin. With the prolongation of boiling time, the flavor change decreased until the limit value was reached after 30 min. The spatial regions of these samples showed that the flavor substance migration from tilapia skin to soup were: 0–10 min period > 10–30 min period > 30–60 min period, which was consistent with the results of content changes (Fig. 1). The volatile components of tilapia skin soup changed with the prolongation of boiling time. There was a certain distance between the fish skin soup groups of 10 min, 30 min and 60 min. The distance between the fish skin soup at 60 min and the fish skin soup at 10 min is the farther. The volatile substances in the fish skin soup at 60 min and the fish skin soup at 10 min were quite different, indicating that the substances in the fish skin was dissolved into the ultrapure water during the boiling process, which made the fish skin soup had a unique taste. However, the distance between 30 min fish skin soup and 60 min fish skin soup was close, indicating that the flavor of fish skin soup changed less after 30 min. Radar graphs (Figs. 2B and 2D) further confirmed the conclusion from PCA analysis. Moreover, their differences for both tilapia skin and tilapia skin soup were mainly detected by metal oxide semiconductors of P40/1 (sensitive to gas with strong oxidation ability such as chloride and fluoride), PA/2 (sensitive to organic compounds and toxic gas such as ethanol, ammonia and amine compounds), and P10/1 (sensitive to combustible gas such as carbon oxygen compound, ammonia and chlorine). The L-type sensors showed almost no response values. P-type and T-type sensors showed relatively high response values. It suggested tilapia skin and tilapia skin soup had rich carbon oxides, polar organic compounds, and certain benzene ring substances.

E-tongue technique was applied to detect the changes of tastes during the boiling process (Fig. 2E–H, and Supplementary Tables 4–5). PCA plots were used to show the tastes of tilapia skin and skin soup samples at different time points, as shown in Figs. 2E and G. The first two components (PC1 was 57.5% and PC2 was 36.6%) explained that 94.1% of the total variance for tilapia skin (Fig. 2E). The two-dimensional intercept size order of the raw and cooked fish skins was as follows: 60 min > 30 min > 10 min. At the same time, the fish skin at 60 min and the skin at 30 min overlapped. Therefore, the taste of the fish skin had changed during the boiling process. Moreover, after 30 min, the change of taste difference becomes less. The first two components (PC1 was 54.3% and PC2 was 42.5%) explained that 96.8% of the total variance for tilapia skin soup (Fig. 2G). The results suggested that the taste of fish skin soup changed during the boiling process. Moreover, the two-dimensional intercept size between fish skin soup at 60 min and fish skin soup at 30 min was the smaller, which indicated that the difference in taste became less after cooking 30 min. The spatial regions of these samples showed that the taste substance migration from tilapia skin to soup were: 0–10 min period > 10–30 min period > 30–60 min period, which was consistent with the results of content changes (Fig. 1) and E-nose analyses (Fig. 2A–D and Supplementary Tables 2–3). Radar graphs (Fig. 2F and H) further confirmed the conclusion from PCA analysis. Seven types of tastes showed similar response value relationships for both tilapia skin and tilapia skin soup at all the time points: bitterness > richness-A > richness-B > saltiness > umami > sweetness > sourness. Both tilapia skin and tilapia skin soup did not show obvious sourness. Five types of tastes (richness-A, saltiness, umami, richness-B, and bitterness) showed no obvious changes for both tilapia skin and tilapia skin soup during the boiling process. Sweetness showed slight changes for both tilapia skin (increase at 0–30 min and then decrease at 30–60 min) and tilapia skin soup (decrease at 10–30 min and then no obvious change at 30–60 min).

### Metabolomics statistical analyses of tilapia skin and soup in the boiling process

The metabolites in tilapia skin and soup were detected by the combination of gas chromatography-time-of-flight-mass spectrometry (GC-TOF-MS) and ultra-high performance liquid chromatography-mass spectrometry/mass spectrometry (UHPLC-MS/MS), and then analyzed by metabolomics techniques. The results showed all the raw and boiled tilapia skins had 783 chemicals (29 were unknown, 155 were analytes, 33 were empty, 566 were metabolites in the “compound name” column) and all the boiled tilapia skin soups had 761 chemicals (34 were unknown, 148 were analytes, 29 were empty, 550 were metabolites in the “compound name” column). The metabolomics statistical analyses of 783 chemicals in tilapia skins and 761 chemicals in tilapia skin soups were used for subsequent PCA and orthogonal partial least-squares to latent structures-discriminate analysis (OPLS-DA) analyses.

**Fig. 3:**
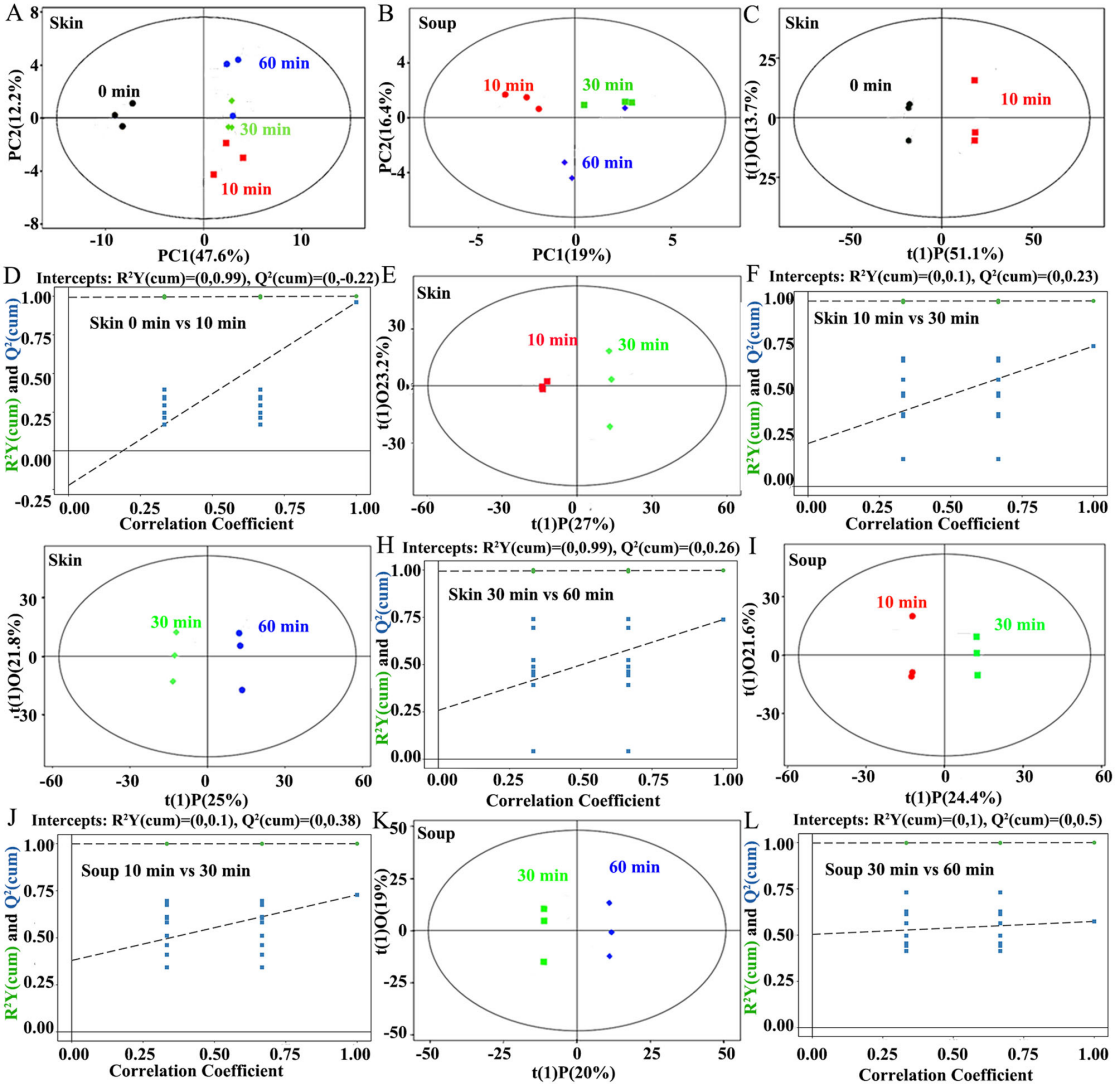
Metabolomics statistical analyses of tilapia skin and soup in the boiling process. **A** PCA score scatter plot of tilapia skin. **B** PCA score scatter plot of tilapia skin soup. **C**, **D** Score scatter plot (**C**) and permutation test (**D**) of OPLS-DA model for the tilapia skins at 0 and 10 min. **E**, **F** Score scatter plot (**C**) and permutation test (**D**) of OPLS-DA model for the tilapia skins at 10 and 30 min. **G**, **H** Score scatter plot (**G**) and permutation test (**H**) of OPLS-DA model for the tilapia skins at 30 and 60 min. **I**, **J** Score scatter plot (**I**) and permutation test (**J**) of OPLS-DA model for the tilapia skin soups at 10 and 30 min. **K**, **L** Score scatter plot (**K**) and permutation test (**L**) of OPLS-DA model for the tilapia skin soups at 30 and 60 min.

PCA plots were used to show the metabolites differences of tilapia skin and skin soup samples at different time points, as shown in Figs. 3A and 3B. Each scatter point represented a sample, the color and shape of the scatter points represented different groupings and we could see that the samples were all within the 95% Hotelling’s T-squared ellipse (big black ellipse in images). The first two components (PC1 was 47.6% and PC2 was 12.2%) explained that 59.8% of the total variance for tilapia skin (Fig. 3A). The first two components (PC1 was 19% and PC2 was 16.4%) explained that 35.4% of the total variance for tilapia skin soup (Fig. 3B). Therefore, the boiling time had a significant effect on the fish skin and fish skin soup. The spatial regions of these samples showed that the metabolite migration from tilapia skin to soup were: 0–10 min period > 10–30 min period > 30–60 min period, which was consistent with the results of content changes (Fig. 1), E-nose analyses (Fig. 2A–D and Supplementary Tables 2–3), and E-tongue analyses (Fig. 2E–H and Supplementary Tables 4–5).

OPLS-DA was applied to analyze the first principal component to identify metabolites that had significant deviation^20^. In the OPLS-DA results (Fig. 3C–L), the individual score scatters plot for all the samples were within the 95% Hotelling’s T-squared ellipse. Compared with the PCA model, the OPLS-DA model can better distinguish tilapia skin and fish skin soup at different cooking times. The samples in each OPLS-DA result showed significant differences (Fig. 3C–L). Moreover, the values of t(1)O were nearly 20.0% for all the OPLS-DA results, which suggested the experiments had good parallelism and repeatability. The comparison between raw and boiled tilapia skin at 10 min (Fig. 3C) showed significant higher t(1) *P*-value than other comparisons (Figs. 3E and 3G), which also confirmed that the metabolite migration from tilapia skin to soup was: 0–10 min period > 10–30 min period > 30–60 min period, which was consistent with the results of content changes (Fig. 1), E-nose analyses (Fig. 2A–D and Supplementary Tables 1–3), E-tongue analyses (Fig. 2E–H and Supplementary Tables 4–5), and PCA analyses of metabolite migration (Fig. 3A, B).

Permutation tests of OPLS-DA models were done to for the statistical analyses. The original model R^2^Y was very close to 1 (Figs. 3D, F, H, J, and L), indicating that the established models conformed to the real situation of the samples. The *Q*^2^ of the stochastic models gradually decreased. In general, the original models could well explain the differences between the two groups of samples^21^. These results showed that the OPLS-DA models were relatively robust and did not have the overfitting problem.

### Differential metabolite analyses of tilapia skin and soup in the boiling process

The obtained chemicals (783 for tilapia skin and 761 for tilapia skin soup) were analyzed by univariate analysis (UVA) to obtain differential chemicals and metabolites of tilapia skin and soup between different time points (0–10, 10–30, and 30–60 min) in the boiling process. The detailed differential metabolites were obtained (Supplementary Tables 6–7). As shown in Supplementary Table 6, the identified differential metabolites (285) in tilapia skin samples included organic oxygen compounds (8), organic acids and derivatives (48), lipids and lipid-like molecules (127), organoheterocyclic compounds (27), organic nitrogen compounds (4), homogeneous non-metal compounds (2), benzenoids (15), nucleosides, nucleotides, and analogs (8), organooxygen compounds (6), phenylpropanoids and polyketides (2), organonitrogen compounds (2), alkaloids and derivatives (3), and others (33). As shown in Supplementary Table 7, the identified differential metabolites (57) in tilapia skin soup samples included organic oxygen compounds (5), organic acids and derivatives (10), lipids and lipid-like molecules (16), organoheterocyclic compounds (10), and others (16).

**Fig. 4:**
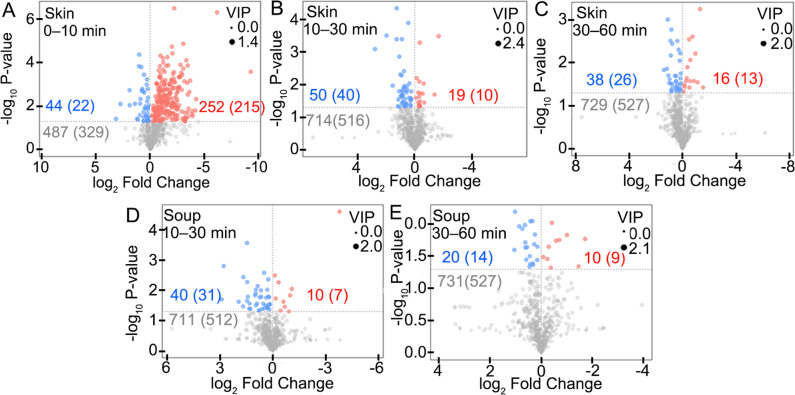
Volcanic maps of the identified differential metabolites of tilapia skins and soups in the boiling process. **A** Tilapia skins at 0 and 10 min. **B** Tilapia skins at 10 and 30 min. **C** Tilapia skins at 30 and 60 min. **D** Tilapia skin soups at 10 and 30 min. **E** Tilapia skin soups at 30 and 60 min. Blue, red, and gray numbers/spots indicate the identified chemicals with significant increases, significant decreases, and no significant changes. The numbers before and in the brackets indicate the numbers of the identified chemicals and metabolites, respectively.

**Fig. 5:**
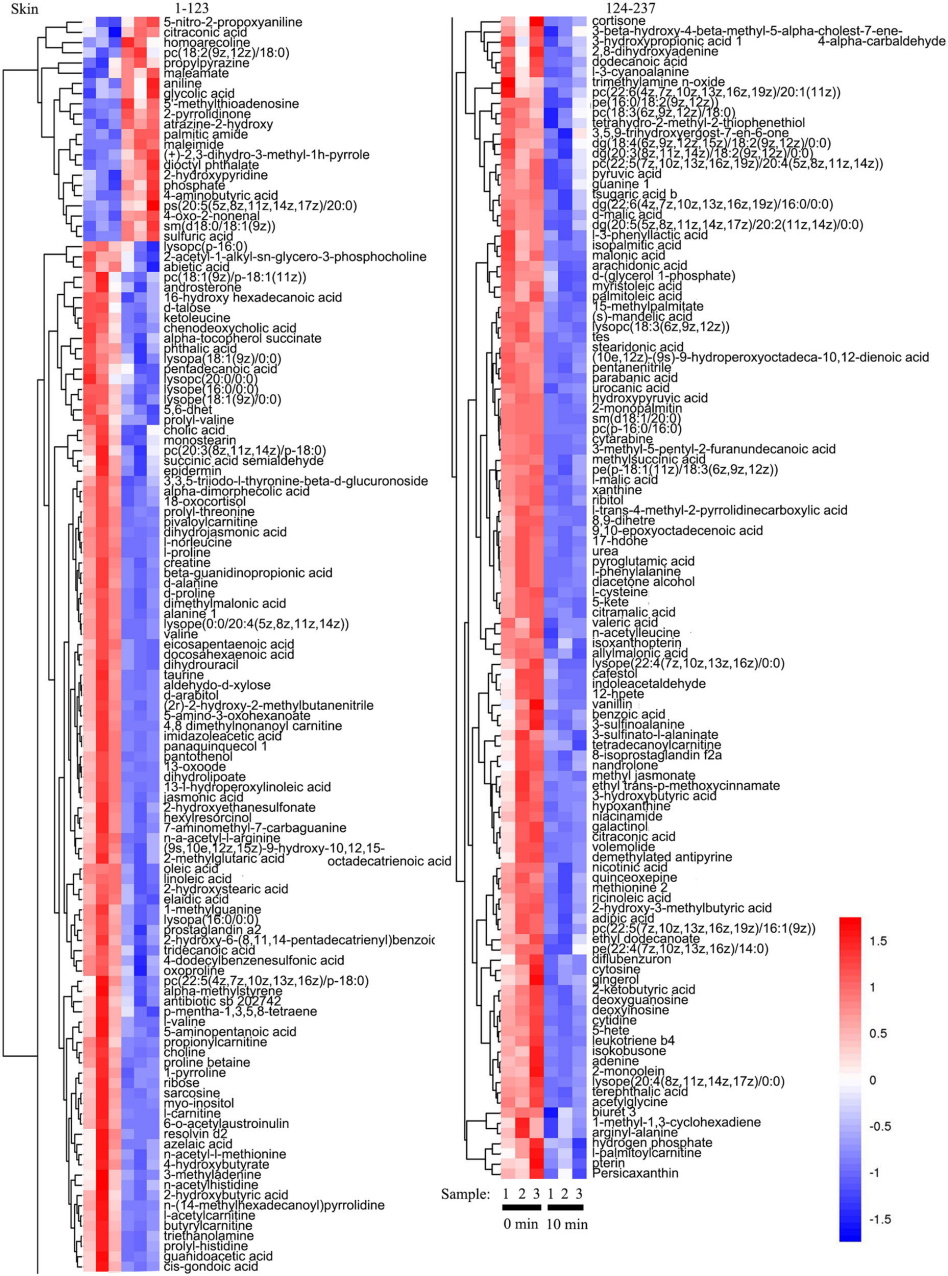
Cluster heat map of the obtained differential metabolites of the tilapia skins at 0 and 10 min in the boiling process. Left image shows No. 1–123 metabolites. Right image shows No. 124–237 metabolites.

**Fig. 6:**
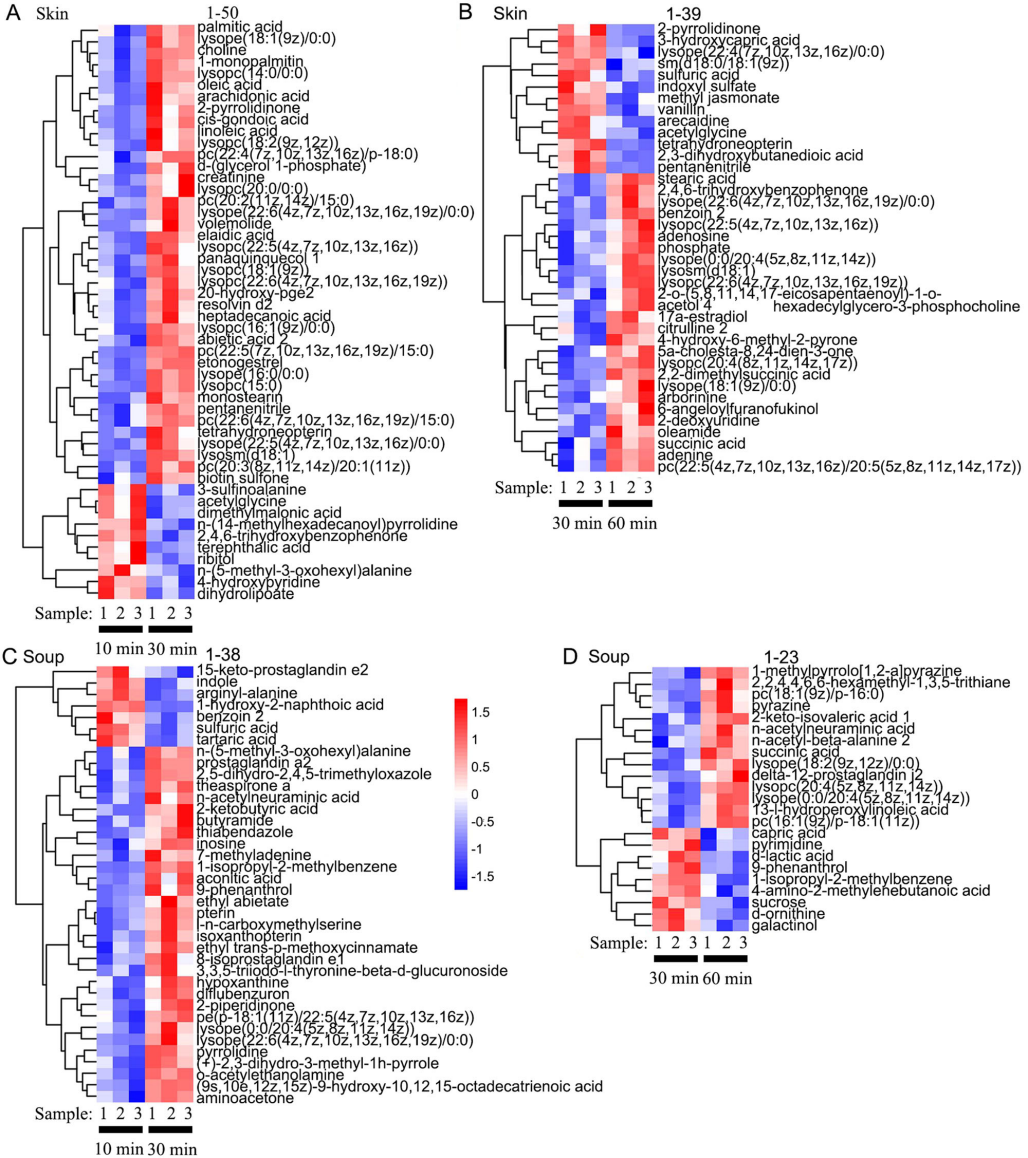
Cluster heat maps of the identified differential metabolites of tilapia skins and soups in the boiling process. **A** Tilapia skins at 10 and 30 min. **B** Tilapia skins at 30 and 60 min. **C** Tilapia skin soups at 10 and 30 min. **D** Tilapia skin soups at 30 and 60 min.

As shown in Fig. 4, the numbers of the identified chemicals and metabolites were shown before and in the brackets such as 44 (22), respectively. In the 0–10 min period, 44 (22) increased, 252 (215) decreased, 487 (329) showed no obvious changes in the tilapia skin (Fig. 4A). In the 10–30 period, 50 (40) increased, 19 (10) decreased, 714 (516) showed no obvious changes in the tilapia skin (Fig. 4B), meanwhile 40 (31) increased, 10 (7) decreased, 711 (512) showed no obvious changes in the tilapia skin soup (Fig. 4C). In the 30–60 period, 38 (26) increased, 16 (13) decreased, 729 (527) showed no obvious changes in the tilapia skin (Fig. 4D), meanwhile 20 (14) increased, 10 (9) decreased, 711 (512) showed no obvious changes in the tilapia skin soup (Fig. 4E). The detailed differential metabolites between different time points were further showed in the cluster heat maps (Figs. 5–6). These results quantitatively demonstrated that the metabolites migration from tilapia skin to soup mainly occurred within 10 min of boiling, which was consistent with the results of content changes (Fig. 1), E-nose analyses (Fig. 2A–D and Supplementary Tables 2–3), E-tongue analyses (Fig. 2E–H and Supplementary Tables 4–5), PCA analyses (Fig. 3A, B), and OPLS-DA analyses (Fig. 3C–L).

Some differential metabolites have been reported to be associated with food flavors. Oleic acid was be positively correlated with juiciness, tenderness, and flavor of black-pig^22^. It (Supplementary Table 6, No. 81) increased in the tilapia skin after boiling in this work. Linoleic acid was associated with fried-food flavor^23^. In the tilapia skin, it (Supplementary Table 6, No. 169) decreased in the 0–10 min period and then decreased in the 10–30 min period. Propylpyrazine was commonly recognized as the flavor source of green, nutty, roasted, hot milk odorants in wheat flour and lupin protein isolate-enriched bread samples^24^. It (Supplementary Table 6, No. 196) increased in the tilapia skin after boiling. Benzoin 2 had an odor of camphor according to Pubchem (an open chemistry database at the National Institutes of Health, https://pubchem.ncbi.nlm.nih.gov/). In this work, benzoin 2 (Supplementary Table 6, No. 280; Supplementary Table 7, No. 46) increased in the 30–60 min period for the tilapia skin and increased in the 10–30 min period for the tilapia skin soup. Further analysis is necessary to analyze the flavor of these differential metabolites.

As shown in E-tongue results (Figs. 2F and 2H), sweetness showed slight changes for both tilapia skin (increase at 0–30 min and then decrease at 30–60 min) and tilapia skin soup (decrease at 10–30 min and then no obvious change at 30–60 min). Typical sweet taste-related compounds include monosaccharides, disaccharides, polyols, D-amino acids, proteins, and synthetic non-nutritive sweeteners^25^. In addition, the loss of bitterness-related compounds such as creatine might lead to the increased sweetness of stewed beef juice^26,27^. Therefore, the sweetness changes might be resulted from the changes of the differential metabolites.

Some differential metabolites have been reported to be associated with food tastes. 5-Nitro-2-propoxyaniline was sweeter with 4000-times intensity than sucrose^28^. After boiling, it (Supplementary Table 6, No. 231) increased in the tilapia skin. d-Alanine was described as a source of sweet^29^. After boiling, it (Supplementary Table 6, No. 22) decreased in the tilapia skin. Valine was described as a source of bitter taste^30^. After boiling, it (Supplementary Table 6, No. 266) decreased in the tilapia skin. Creatine could be converted into creatinine after boiling and could show bitter taste together with creatinine^31^. After boiling, it (Supplementary Table 6, No. 16) decreased in the tilapia skin. N-acetylglycine was described as a source of bitter taste^32^. After boiling, it (Supplementary Table 6, No. 24) decreased with time in the tilapia skin. Further analysis is necessary to analyze the tastes of these differential metabolites.

### Effects of different metabolites on the taste and nutritional content of tilapia skin

**Fig. 7:**
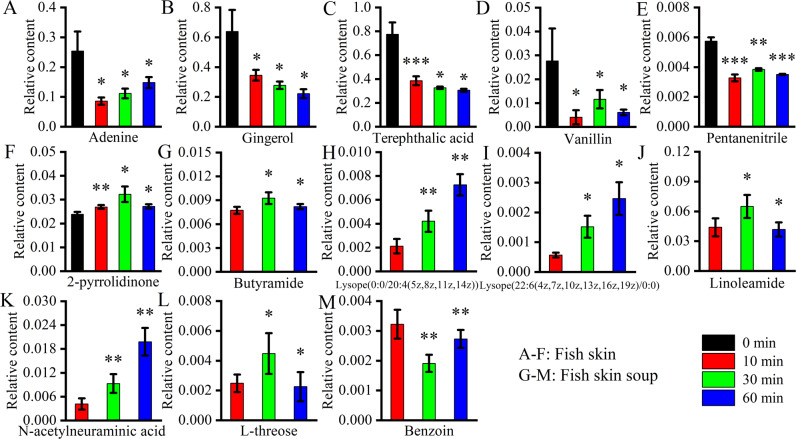
The relative contents of differential metabolites during the processing with VIP > 1 (*p < 0.05, **p < 0.01, ***p < 0.005, compared with the sample at the last designated time point). **A**–**F** Tilapia skin. **G**–**M** Tilapia skin soups. The error bars represent standard deviation (*n* = 3).

Based on the OPLS-DA models, 6 chemicals (Fig. 7A–F) were screened out in the differential metabolites of tilapia skin with Variable Importance in the Projection (VIP) > 1 and *p* < 0.05. Adenine is a small molecule compound produced by the continuous degradation of nucleic acid. It would be further degraded into the human body through the diet to produce uric acid^33^. Uric acid could cause gout^34^. As shown in Fig. 7A, adenine in fish skin was greatly reduced after cooking for 10 min, so the boiling could reduce the adenine content, making the fish skin healthier^35^. As shown in Fig. 7B, the content of gingerol in the fish skin decreased continuously during the boiling process. Some studies have shown that gingerol could be used as a condiment to increase the salty and umami taste of food^36^, but gingerol itself had spicy and bitter taste^37^. Therefore, there would be a certain bitter umami and saltiness in the tilapia skin. Under certain conditions, terephthalic acid could be converted into vanillin^38^, which was a compound with a unique taste of vanilla. During the boiling process of fish skin, the content of terephthalic acid (Fig. 7C) decreased significantly. In addition, the content of vanillin (Fig. 7D) increased significantly after boiling for 30 min, but decreased after boiling for 60 min. Therefore, the boiling time should not be too long to make the fish skin a better taste^38^. Compared with other compounds, the content of pentanenitrile (Fig. 7E) in fish skin was less, which might have little effect on the flavor. The presence of 2-pyrrolidinonede might reduce the bitterness and astringency in the food^39^. The 2-pyrrolidinonede content (Fig. 7F) of fish skin increased significantly during the process of boiling, and reached the peak value when boiling for 30 min. The astringency and bitterness of the fish skin might also change to a certain extent after boiling for 30 min. Therefore, the boiling time for fish skin should not be too long and 10 min might be the best time point among these three time points.

### Effects of different metabolites on the taste and nutritional content of tilapia skin soup

Based on the OPLS-DA models, seven chemicals (Fig. 7G–M) were screened out in the differential metabolites of tilapia skin soup with VIP > 1 and *p* < 0.05. Butyramide had nutty flavor and its content (Fig. 7G) in the fish skin soup increased significantly when the tilapia skin was boiled for 30 min, which might contribute to the flavor of the tilapia skin soup. Both lysope(0:0/20:4(5z,8z,11z,14z)) and lysope(22:6(4z,7z,10z,13z,16z,19z)/0:0) are lysophospholipids. During the boiling process, the contents of these compounds (Figs. 7H, I) in fish skin soup increased significantly. As far as we know, the phospholipids had a certain influence on the flavor of food^21^, so lysope(0:0/20:4(5z,8z,11z,14z)) and lysope(22:6(4z,7z,10z,13z,16z,19z)/0:0) had significant contribution to the flavor of fish skin soup. Linoleic acid was an endogenous lipid that was believed to be involved in various life activities such as fetal growth and development^40^. After boiling for 30 min, the content of linoleic acid (Fig. 7J) in fish skin soup was the highest. N-acetylneuraminic acid was a natural product with antioxidant effects. Studies have shown that it had a certain effect on the flavor of camel milk^41^. During the boiling process of fish skin, the N-acetylneuraminic acid content (Fig. 7K) of fish skin soup increased significantly, which might have some influences on the flavor of fish skin soup. L-threose was a significant degradation product of ascorbic acid in the presence of oxygen^42^. After boiling for 30 min, the L-threose content (Fig. 7L) is the highest. Benzoin had an odor of camphor according to Pubchem. Benzoin content (Fig. 7M) increased in the 10–30 min period for the tilapia skin soup.

### Analyses of metabolic pathways

**Fig. 8:**
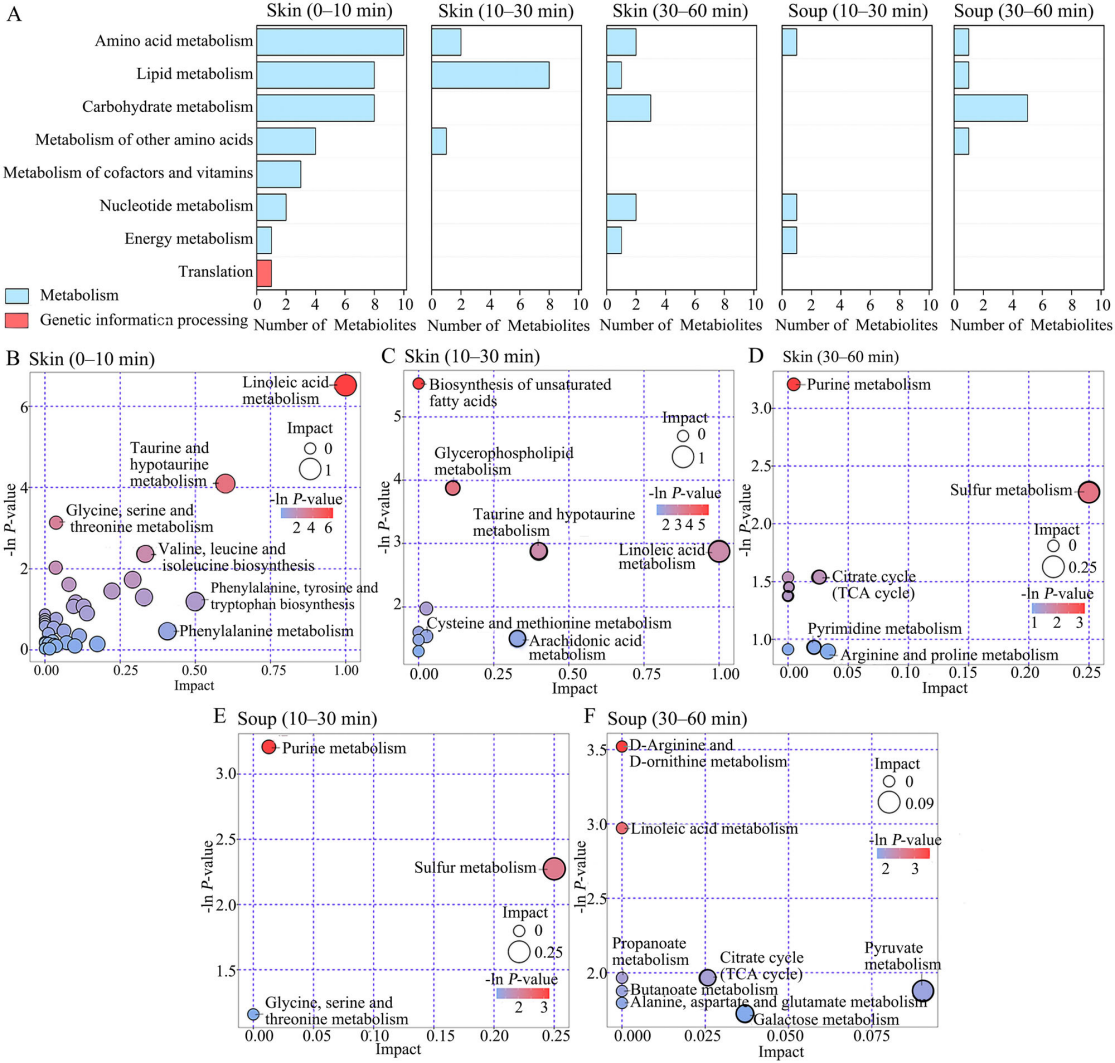
Metabolic pathway analysis of tilapia skin and soup in the boiling process. **A** KEGG pathway annotation of differential metabolites between different samples (tilapia skins at 0 and 10 min, tilapia skins at 10 and 30 min, tilapia skins at 30 and 60 min, tilapia skin soups at 10 and 30 min, and tilapia skin soups at 30 and 60 min). **B**–**F** Bubble plots of differential metabolites between different samples. The larger the circle size and the deeper the red color intensity, the greater the impact on the respective pathway. **B** Tilapia skins at 0 and 10 min. **C** Tilapia skins at 10 and 30 min. **D** Tilapia skins at 30 and 60 min. **E** Tilapia skin soups at 10 and 30 min. **F** Tilapia skin soups at 30 and 60 min.

The metabolic pathways of the obtained differential metabolites data were analyzed in this section. The obtained results were shown in Kyoto Encyclopedia of Genes and Genomes (KEGG) pathway annotation images (Fig. 8A) and bubble plots (Fig. 8B–F). For the tilapia skin 0–10 min group, the differential metabolites were primarily involved in “amino acid metabolism”, “lipid metabolism”, “carbohydrate metabolism”, “metabolism of other amino acids”, “metabolism of cofactors and vitamins” (Fig. 8A). Moreover, the differential metabolites were also involved in translation, a type of genetic information processing pathway. The “linoleic acid metabolism”, “taurine and hypotaurine metabolism”, and “glycine, serine and threonine metabolism” were significantly different in this tilapia skin 0–10 min group (Fig. 8B). For the tilapia skin 10–30 min group, the differential metabolites were primarily involved in lipid metabolism (Fig. 8A), and the “biosynthesis of unsaturated fatty acids” and “glycerophospholipid metabolism” were significantly different (Fig. 8C). For the tilapia skin 30–60 min group, the differential metabolites were primarily involved in “carbohydrate metabolism” (Fig. 8A), and the “purine metabolism” were significantly different (Fig. 8D). For the tilapia skin soup 10–30 min group, the differential metabolites were not primarily involved in any metabolism (Fig. 8A), and the “purine metabolism” was significantly different (Fig. 8E). For the tilapia skin soup 30–60 min group, the differential metabolites were primarily involved in “carbohydrate metabolism” (Fig. 8A), and the “D-arginine and D-ornithine metabolism” were significantly different (Fig. 8F). These metabolic pathways might provide information to understand the effect of boiling processing on the chemical changes in tilapia skin boiling process.

According to the results in KEGG pathway annotation images (Fig. 8A) and bubble plots (Fig. 8B–F). Metabolism pathways were: 0–10 min period > 10–30 min period > 30–60 min period, which was consistent with the results of content changes (Fig. 1), E-nose analyses (Fig. 2A–D and Supplementary Tables 2–3), E-tongue analyses (Fig. 2E–H and Supplementary Tables 4–5), PCA analyses of metabolites (Fig. 3A, B), OPLS-DA analyses of metabolites (Fig. 3C–L), and metabolites migration from tilapia skin to soup (Figs. 4–7). Especially, genetic information processing was only involved in the initial 10 min (Fig. 8). Therefore, the initial 10 min was the key time for the metabolite changes in the boiling process of tilapia skin.

In this study, the sensory and metabolites migration in the boiling process of tilapia skin were analyzed. The results suggested content changes, flavor changes, taste changes, and metabolite changes for both tilapia skin and soup mainly happened in 30 min. Moreover, the sensory and metabolite migration from tilapia skin to soup was fast firstly (0–10 min) and then slow (10–60 min). The boiling time for fish skin should not be too long and 10 min might be the best time point among these three time points (10, 30, and 60 min). The differential metabolites (6 and 7 for tilapia skin and soup, respectively, with VIP > 1 and *p* < 0.05) were screened out in the boiling process. This work would be beneficial to understand how the substances migrate from the tilapia skin to the soup in the boiling process. It also provided a detailed metabolomics method to analyze metabolites migration in foods. These metabolic pathways might provide information to understand the effect of processing on the small molecular chemical changes in foods. Finally, this work would also be beneficial for the value-added utilization of aquatic by-product fish skin. However, it should be noted that the detailed reaction mechanism and metabolites change did not be analyzed due to the weakness of E-nose, E-tongue, and untargeted metabolomics. Further works are required to analyze the flavor, tastes, and metabolite change routes of the differential metabolites.

## Methods

### Materials and reagents

In Tongwei (Hainan) Aquatic Food Co., Ltd., the skins with scales of Genetic Improvement of Farmed tilapia after fillet processing were frozen and then the frozen skins with scales were shipped by cold chain and stored at −18 °C in our laboratory. The used chemicals in this work were: methanol (high-performance liquid chromatography (HPLC) grade, CNW Technologies GmbH, Düsseldorf, Germany), acetonitrile (HPLC grade, CNW Technologies), chloroform (HPLC grade, Adamas-beta Reagent Co., Ltd., Shanghai, China), pyridine (HPLC grade, Adamas-beta), methoxyamine hydrochloride (analytical grade (AR), Tokyo Chemical Industry Co., Ltd., Tokyo, Japan), adonitol (purity ≥ 99%, Sigma-Aldrich, Shanghai, China), ammonium acetate (HPLC grade, Sigma-Aldrich), ammonium hydroxide (HPLC grade, Fisher Chemical, Fair Lawn, NJ, USA), Bis(trimethylsilyl)trifluoroacetamide with 1% (v/v) trimethylsilyl chloride (REGIS Technologies Inc., Chicago, IL, USA), and fatty acid methyl esters (Dr. Ehrenstorfer Gmbh, Augsburg, Germany).

### Tilapia skin boiling process

Frozen tilapia skins with scales were naturally thawed at room temperature. The scales and fats were removed. After cleaning, the tilapia skins were cut into pieces (5 × 6 cm). Then, 2 g of tilapia skin was mixed with 10 mL of ultrapure water in a glass vial with cap. Finally, the glass vial was heated in 100 °C water bath for 10, 30, and 60 min. At the designated time points, the tilapia skin and soup were carefully separated for subsequent measurements.

### Content analysis of tilapia skin and soup

#### Mass loss ratio

The mass loss ratios of tilapia skin and soup were calculated according to below equation:

1 $$Mass\;loss\;ratio(g/100g) = \frac{{m1 - m2}}{{m1}} \times 100$$

whereas *m*1 is the initial mass of tilapia skin (2 g) or tilapia soup (10 g), *m*2 is the measured mass at different time points (10, 30, and 60 min).

#### Moisture content of tilapia skin

Moisture contents of tilapia skin samples were measured according to Chinese national standard “Determination of moisture content in foods” (GB 5009.3-2016). Briefly, the tilapia skin samples were weighed, dried at 101–105 °C, and weighed. The moisture content (g/100 g of tilapia skin) was calculated by dividing the removed moisture mass by the tilapia skin sample mass and multiplying by 100.

#### Ash content

Protein contents of tilapia skin and soup samples were measured according to Chinese national standard “Determination of ash content in foods” (GB 5009.4-2016). Briefly, the samples were weighed, dried at 550 ± 25 °C, and weighed. The ash content (g/100 g of sample) was calculated by dividing the ash mass by the sample mass and multiplying by 100.

#### Soluble solid content of tilapia skin soup

Briefly, the tilapia skin soup samples were weighed, dried at 101–105 °C, and weighed. The soluble solid content (g/100 g of tilapia skin soup) was calculated by dividing the dried solid content mass by the tilapia skin soup mass and multiplying by 100.

#### Protein content

Protein contents of tilapia skin and soup samples were measured according to Chinese national standard “Determination of protein content in foods” (GB 5009.5-2016). Briefly, the samples were weighted, catalytically digested, and detected by Kjeltec 8400 protein analyzer (FOSS, Hillerød, Denmark). The protein content (g/100 g of sample) was calculated by multiplying the nitrogen content by 6.25.

### Sensory analysis of tilapia skin and soup

#### E-nose analysis

Tilapia skin or soup samples (*n* = 3) were examined by Fox 4000 sensory array fingerprint analyzer (Alpha M.O.S., Toulouse, France) with 18 metal oxide semiconductors (LY2/LG, LY2/G, LY2/AA, LY2/GH, LY2/gCTL, LY2/gCT, P10/1, P10/2, P40/1, P30/1, P40/2, P30/2, PA/2, T30/1, T70/2, T40/2, T40/1, and TA/2: Supplementary Table 1) to sense different chemicals^43,44^. The obtained data were input into Origin 2018 software (OriginLab Corp., Northampton, MA, USA) for PCA and Radar chart analysis. One-way ANOVA followed by Duncan’s test was used for statistical comparisons.

#### E-tongue analysis

Tilapia skin or soup samples (*n* = 3) were mixed with 25 mL of ultrapure water, homogenized, sonicated, and centrifuged (10614 × *g*) for 15 min at 4 °C. The oil layer was removed and then the supernatant was filtered to 100 mL quantitative bottles. The precipitates were treated by this process twice and filtered to the 100 mL quantitative bottles. Subsequently, ultrapure water was added to the quantitative bottles to ensure the liquid volume was 100 mL. Finally, 5 mL of the samples were analyzed by ASTREE liquid and taste analyzer (Alpha M.O.S) at a measurement time of 120 s^45^. The obtained data were input into Origin 2018 software (OriginLab Corp.) for PCA and Radar chart analysis. One-way ANOVA followed by Duncan’s test was used for statistical comparisons.

### Metabolomics experiments

#### Extraction of metabolites from tilapia skin and soup

Tilapia skin and soup samples were immediately frozen in liquid nitrogen and then stored in −80 °C freezer (Forma 900 series, Thermo Fisher Scientific, Rockford, IL, USA) for subsequent analysis. The frozen skin samples were sheared and mixed with stainless steel beads and liquid nitrogen in a grinding mortar for 10 min. The frozen samples were grinded for 60 s at 60 Hz in a Tissuelyser-24 automatic samples rapid homogenizer (Shanghai Jingxin Technology Co. Ltd., Shanghai, China). The frozen soup samples were heated at 50 °C for 10 min.

Skin (100 ± 1 mg) or soup (100 μL) samples were moved into 2 mL of Eppendorf tubes and 1 mL of precooled extract solvent (mechanol:acetonitrile:water = 2:2:1, volume ratio) with internal standard (10 μL, 10 μg/mL, clenbuterol for positive ion mode and chloramphenicol for the negative ion mode analyses) was added^46^. After adding stainless steel beads, the mixtures were grinded for 4 min in a JXFSTPRP-24 grinder (Shanghai Jingxin Technology Co. Ltd.) and ultrasonicated for 5 min in ice. After repeating the ultrasonication process twice, the samples were centrifuged (13800 × *g*) for 15 min at 4 °C and were sat quietly for 1 h at −40 °C. Then, 70 μL of supernatant was used for subsequent UHPLC (Vanquish, Thermo Fisher Scientific, Waltham, MA, USA)-MS/MS (Q Extractive HFX, Thermo Fisher Scientific) analysis and the mixtures of equal volumes of all the samples were used as quality-control (QC) samples. In the meantime, 200 μL of supernatant was concentrated in a vacuum concentrator. Then, 30 μL of methoxyamine hydrochloride (20 mg/mL in pyridine) was added and incubated at 80 °C for 30 min. Subsequently, 40 μL of bis-(trimethylsilyl)-trifluoroacetamide with 1% (v/v) trimethylsilyl chloride was added and incubated at 70 °C for 1 h for subsequent GC (7890 A, Agilent Technologies Inc., CA, USA)-TOF-MS (Pegasus HT, LECO Corp., San Jose, CA, USA) analysis and the mixtures of equal volumes of all the samples with 5 μL of saturated fatty acid methyl ester (in chloroform) were used as QC samples.

#### UHPLC-MS/MS

UHPLC-MS/MS experiments were performed by a UHPLC system (Vanquish, Thermo Fisher Scientific, Waltham, MA, USA) with a UPLC BEH Amide column (2.1 × 100 mm, particle size 1.7 μm, J&W Scientific, Folsom, CA, USA) coupled to Q-Exactive HFX MS (Orbitrap MS, Thermo Fisher Scientific, Waltham, MA, USA)^47^. The mobile phase consisted of 25 mmol/L ammonium acetate and 25 mmol/L ammonia hydroxide in water (pH = 9.75) (A) and acetonitrile (B). The flow rate was 500 μL/min. The auto-sampler temperature was 4 °C and the injection volume was 3 μL. The gradient setting: 0~0.5 min, 95% B; 0.5~7 min, 95%~65% B; 7~8 min, 65%~40% B; 8~9 min, 40% B; 9~9.1 min, 40%~95% B; 9.1~12 min, 95% B.

The QE HFX MS was used due to its ability to acquire MS/MS spectra on information-dependent acquisition (IDA) mode in the control of the acquisition software (Xcalibur 4.1, Thermo Fisher Scientific, Waltham, MA, USA). In this mode, the acquisition software continuously evaluates the full scan MS spectrum. The electrospray ionization (ESI) source conditions were set as following: sheath gas flow rate as 30 Arb, Aux gas flow rate as 25 Arb, capillary temperature 350 °C, full MS resolution as 60000, MS/MS resolution as 7500, collision energy as 10/30/60 in Na^+^/Ca^2+^ exchanger (NCE) mode, spray voltage as 3.6 kV (positive) or −3.2 kV (negative), respectively.

#### GC-TOF-MS

GC-TOF-MS experiments were performed using an 7890 A (Agilent, Palo Alto, CA, USA) GC-TOF-MS with Chroma TOF version 4.51 software (LECO Corp.)^20^. The system utilized a DB-5MS (30 m × 250 μm × 0.25 μm) capillary column (Agilent). Briefly, 1 μL aliquot of sample was injected in splitless mode. Helium was used as the carrier gas. The front inlet septum purge flow was 3 mL/min. The gas flow rate through the column was 1 mL/min. The initial temperature was kept at 50 °C for 1 min, then raised to 310 °C at a rate of 10 °C per min, and then kept at 310 °C for 8 min. The front injection, transfer line, and ion source temperatures were 280, 280, and 250 °C, respectively. The energy was −70 eV in electron impact mode. The MS data were acquired in full-scan mode with the m/z range of 50–500 at a rate of 12.5 spectra per second after a solvent delay of 6.27 min.

### Metabolomics statistical analysis

#### Metabolomics data management and preprocessing

The raw data were managed as follows^48^: The deviation values were filtered for all the peaks to remove noise based on relative standard deviation. Then, all the peaks were filtered to retain the peak with the null value of <50% for a single group or for all the groups. The missing values were filled with median values. The data were normalized by the internal standard method to obtain the relative quantitative values.

The managed UHPLC-MS/MS data were converted to mzXML format using the ProteoWizard software (ProteoWizard Software Foundation, Palo Alto, CA, USA)^49^ and then treated by R package XCMS (V3.6.3, Lucent Technologies, Jasmine Hill, NJ, USA) for peak detection, extraction, alignment and integration^50^. Then, the metabolite annotation was performed using an in-house MS2 database (BiotreeDB version 2.1) of Biotree Biotech Co., Ltd. (Shanghai, China). The cutoff value for annotation was set as 0.3.

The managed GC-TOF-MS data were analyzed by Chroma TOF (V4.3x, LECO Corp., MI, USA) software: peak extraction, baseline adjustment, deconvolution, alignment and integration. The LECO-Fiehn RTX5 database was used for metabolites identification by matching the retention index and the mass spectrum. Finally, the peaks detected in <50% of QC samples or relative standard deviation >30% in the QC samples were discarded^48^.

The pretreated UHPLC-MS/MS and GC-TOF-MS data were merged in R. Studio software and then input into the SIMCA software (V16.0.2, Sartorius Stedim Data Analytics AB, Umea, Sweden) for data merging.

#### PCA

The merged data were treated by logarithmical transformation and center scaling. Then, the data were automatically analyzed by a PCA model and the score scatter plot images were obtained^51^.

#### OPLS-DA

The merged data were treated by logarithmical transformation and unit variance scaling. Then, the first principal component was analyzed by an OPLS-DA model and the score scatter plot images were obtained^51^. A permutation test was performed to validate the OPLS-DA model^18^. In the permutation test, the corresponding OPLS-DA model was established multiple times (*n* = 200) by randomly changing the arrangement order of the categorical variable Y to obtain the R^2^Y and *Q*^2^-values of the random model (permutation test of OPLS-DA model).

### Differential metabolite analysis

#### UVA

UVA was performed to obtain differential metabolites^52^. *P*-value of Student’s *T*-test was set as <0.05. The VIP in the OPLS-DA model was set as >1. The obtained differential metabolites data were used to prepare volcanic maps in the SIMCA software: the abscissa was log_2_ Fold Change and the ordinate was −log_10_ *P*-value.

#### Cluster heat map

The obtained differential metabolites data were input into R. Studio software. The Euclidean distance matrix was calculated and then the data were analyzed by the complete linkage method to obtain cluster heat map^53^. At the same time, the differential compounds present in fish skins or fish skin soups at different stages were screened for statistical analysis.

### Metabolic pathway analysis

The obtained differential metabolites data were input into R. Studio software and were mapped to the corresponding species (*Oreochromis niloticus* Nile tilapia) in KEGG Pathway database and organic small molecule bioactivity database PubChem. After the mapping match, metabolic pathways were search and analyzed in pathway database of the corresponding species (*Danio rerio*). The analyzed data were shown in KEGG pathway annotation images and Bubble plot images^17^.

## Supplementary information

Supplementary Material

## Data Availability

The authors declare that all data supporting the findings of this study are available in the paper and supplementary information.
