# Supplementary material for: Sensory and metabolite migration from tilapia skin to soup during the boiling process: fast and then slow
Source: NPJ Sci Food. 2022 Nov 11;6:52. doi: 10.1038/s41538-022-00168-w (PMC9652418; doi:10.1038/s41538-022-00168-w)
Supplement: Supplementary file 1 — Supplementary Material [file 41538_2022_168_MOESM1_ESM.pdf]

Supplementary Materials:

Sensory and metabolite migration from tilapia skin to soup during the boiling process: Fast and then slow

Jiahui Chen[1,2], Yinghua Nie[1,2], Jiamin Xu[1,2], Shudan Huang[1,2], Jie Sheng[2], Xichang Wang[2], Jian Zhong[1,2,*]

[1]Xinhua Hospital, Shanghai Institute for Pediatric Research, Shanghai Key Laboratory of Pediatric Gastroenterology and Nutrition, Shanghai Jiao Tong University School of Medicine, Shanghai 200092, China

[2]National R&D Branch Center for Freshwater Aquatic Products Processing Technology (Shanghai), Integrated Scientific Research Base on Comprehensive Utilization Technology for By-Products of Aquatic Product Processing, Ministry of Agriculture and Rural Affairs of the People's Republic of China, Shanghai Engineering Research Center of Aquatic-Product Processing and Preservation, College of Food Science & Technology, Shanghai Ocean University, Shanghai 201306, China

[*]Corresponding author at: Xinhua Hospital, Shanghai Institute for Pediatric Research, Shanghai Key Laboratory of Pediatric Gastroenterology and Nutrition, Shanghai Jiao Tong University School of Medicine, Shanghai 200092, China

E-mail: jzhong@shsmu.edu.cn (J. Zhong)

Abbreviated running title: Sensory and metabolite migration from tilapia skin to soup

Supplementary Table 1. Sensitivity of metal oxide semiconductors in E-nose instrument.

No.	Sensor	Properties	Reference materials
1	LY2/LG	Sensitivity for gas with strong oxidation ability	Chlorine, fluorine, nitrogen oxides and sulfides
2	LY2/G	Sensitivity for toxic gas	Ammonia, amine compounds, carbon and oxygen compounds
3	LY2/AA	Sensitivity for organic compounds	Ammonia, ethanol and acetone
4	LY2/GH	Sensitivity for toxic gas	Ammonia and amine compounds
5	LY2 /gCTL	Sensitivity for toxic gas	Hydrogen sulfide
6	LY2/gCT	Sensitivity for inflammable gas	Propane and butane
7	T30/1	Sensitivity for organic compounds	Polar organic compound and hydrogen sulfide
8	P10/1	Sensitivity for combustible gas	Carbon oxygen compound, ammonia and chlorine
9	P10/2	Sensitivity for inflammable gas	Methane and ethane
10	P40/1	Sensitivity for gas with strong oxidation ability	Chlorid and fluoride
11	T70/2	Sensitivity for aromatic compounds	Toluene and xylene
12	PA/2	Sensitivity for organic compounds and toxic gas	Ethanol, ammonia and amine compounds
13	P30/1	Sensitivity for combustible gas and organic compounds	Carbon oxygen compound, combustion product, ammonia and ethanol
14	P40/2	Sensitivity for gas with strong oxidation ability	hydrogen sulfide, chlorine and fluoride
15	P30/2	Sensitivity for organic compounds	Ethanol, combustion products, aldehydes and hydrogen sulfide
16	T40/2	Sensitivity for gas with strong oxidation ability	Chlorid and fluoride
17	T40/1	Sensitivity for gas with strong oxidation ability	Fluoride
18	TA/2	Sensitivity for organic compounds	Ethanol

Supplementary Table 2. E-nose results of tilapia skin detected by metal oxide semiconductors at different time points.

No.	Sensor	0 min	10 min	30 min	60 min
1	LY2/LG	0.0089±0.0008[a]	0.0096±0.0005[a]	0.0092±0.0006[a]	0.0095±0.0005[a]
2	LY2/G	-0.0036±0.0007[a]	-0.0034±0.0004[a]	-0.0048±0.0004[b]	-0.0048±0.0004[b]
3	LY2/AA	-0.0034±0.0004[a]	-0.0030±0.0006[a]	-0.0040±0.0004[a]	-0.0040±0.0007[a]
4	LY2/GH	-0.0044±0.0007[a]	-0.0046±0.0008[a]	-0.0059±0.0002[b]	-0.0057±0.0004[b]
5	LY2/gCTL	-0.0036±0.0004[b]	-0.0023±0.0008[a]	-0.0034±0.0006[a]	-0.0039±0.0008[b]
6	LY2/gCT	-0.0029±0.0011[a]	-0.0044±0.0001[a]	-0.0058±0.0008[b]	-0.0064±0.0003[b]
7	T30/1	0.0699±0.0047[c]	0.0841±0.0017[b]	0.0925±0.0032[a]	0.0939±0.0062[a]
8	P10/1	0.1040±0.0065[b]	0.1266±0.0028[a]	0.1326±0.0087[a]	0.1331±0.0091[a]
9	P10/2	0.0746±0.0045[b]	0.0950±0.0035[a]	0.0998±0.0059[a]	0.0981±0.0072[a]
10	P40/1	0.1187±0.0073[b]	0.1458±1.0032[a]	0.1542±1.0103[a]	0.1539±0.0105[a]
11	T70/2	0.0552±0.0040[b]	0.0678±0.0023[a]	0.0725±0.0028[a]	0.0740±0.0046[a]
12	PA/2	0.1109±0.0073[b]	0.1328±1.0032[a]	0.1418±1.0061[a]	0.1429±0.0094[a]
13	P30/1	0.0466±0.0080[b]	0.0687±0.0075[a]	0.0801±0.0070[a]	0.0810±0.0033[a]
14	P40/2	0.0304±0.0025[c]	0.0389±0.0017[b]	0.0439±0.0018[a]	0.0440±0.0036[a]
15	P30/2	0.0372±0.0084[b]	0.0637±0.0097[a]	0.0735±0.0062[a]	0.0749±0.0031[a]
16	T40/2	0.0365±0.0038[c]	0.0493±0.0032[b]	0.0580±0.0032[a]	0.0571±0.0047[a]
17	T40/1	0.0786±0.0036[b]	0.0991±0.0031[a]	0.0959±0.0036[a]	0.0967±0.0071[a]
18	TA/2	0.1012±0.0045[b]	0.1258±0.0039[a]	0.1209±0.0050[a]	0.1231±0.0086[a]

Different letters in the rows indicate significant differences ($p < 0.05$).

Supplementary Table 3. E-nose results of tilapia skin soup detected by metal oxide semiconductors at different time points.

No.	Sensor	10 min	30 min	60 min
1	LY2/LG	0.0106±0.0015[b]	0.0148±0.0008[a]	0.0132±0.0023[a]
2	LY2/G	-0.0055±0.0010[a]	-0.0082±0.0004[b]	-0.0087±0.0019[b]
3	LY2/AA	-0.0046±0.0003[a]	-0.0064±0.0009[a]	-0.0074±0.0014[b]
4	LY2/GH	-0.0068±0.0005[a]	-0.0095±0.0009[b]	-0.0103±0.0018[b]
5	LY2/gCTL	-0.0036±0.0004[a]	-0.0600±0.0004[b]	-0.0068±0.0016[b]
6	LY2/gCT	-0.0053±0.0006[a]	-0.0066±0.0015[a]	-0.0078±0.0006[b]
7	T30/1	0.0876±0.0014[a]	0.0993±0.0027[b]	0.0968±0.0030[b]
8	P10/1	0.1313±0.0031[a]	0.1478±0.0039[b]	0.1405±0.0050[b]
9	P10/2	0.0997±0.0016[b]	0.1089±0.0057[a]	0.1053±0.0042[a]
10	P40/1	0.1511±0.0031[b]	0.1675±0.0058[a]	0.1620±0.0067[a]
11	T70/2	0.0714±0.0019[b]	0.0803±0.0028[a]	0.0781±0.0028[a]
12	PA/2	0.1413±0.0024[b]	0.1604±0.0051[a]	0.1544±0.0044[a]
13	P30/1	0.0793±0.0085[b]	0.09895±0.0137[a]	0.1025±0.0053[a]
14	P40/2	0.0400±0.0012[b]	0.0454±0.0029[a]	0.0458±0.0017[a]
15	P30/2	0.0743±0.0104[b]	0.0811±0.0168[a]	0.0992±0.0059[a]
16	T40/2	0.0519±0.0031[b]	0.0589±0.0067[a]	0.0629±0.0034[a]
17	T40/1	0.0965±0.0014[b]	0.1029±0.0032[a]	0.0967±0.0034[b]
18	TA/2	0.1217±0.0032[b]	0.1294±0.0036[a]	0.1226±0.0042[a]

Different letters in the rows indicate significant differences ($p < 0.05$).

Supplementary Table 4. E-tongue results of tilapia skin at different time points.

Time (min)	0	10	30	60
Sourness	-389.42±11.64[c]	-368.76±6.82[b]	-362.00±2.99[b]	-365.59±5.73[a]
Richness-A	2421.12±5.67[a]	2420.63±8.10[a]	2409.29±3.09[a]	2423.35±6.50[a]
Saltiness	1584.94±12.77[b]	1604.08±10.83[a]	1598.05±1.21[a]	1605.47±8.41[a]
Umami	1317.13±7.68[a]	1333.73±8.12[b]	1335.07±3.90[b]	1332.69±5.47[b]
Richness-B	1700.32±2.49[c]	1722.00±3.04[b]	1729.14±5.04[a]	1727.29±0.39[a]
Sweetness	629.47±89.36[b]	710.29±85.67[a]	832.87±66.78[a]	710.98±28.55[a]
Bitterness	2488.58±18.23[b]	2508.75±20.00[a]	2508.24±10.27[a]	2503.11±10.90[a]

Different letters in the rows indicate significant differences ($p < 0.05$).

Supplementary Table 5. E-tongue results of tilapia skin soup at different time points.

Time (min)	10	30	60
Sourness	-366.98±6.49[b]	-390.46±9.67[b]	-401.25±18.38[a]
Richness-A	2372.27±6.59[b]	2390.12±5.74[a]	2397.37±3.35[a]
Saltiness	1537.40±23.32[a]	1530.64±16.60[a]	1508.55±20.81[a]
Umami	1296.56±9.76[b]	1288.74±7.96[b]	1276.06±8.62[c]
Richness-B	1699.88±1.97[a]	1688.77±2.28[b]	1687.29±8.89[b]
Sweetness	912.74±60.17[a]	842.59±61.98[a]	846.26±95.09[a]
Bitterness	2458.15±14.69[a]	2444.66±14.17[a]	2427.85±10.92[b]

Different letters in the rows indicate significant differences ($p < 0.05$).

Supplementary Table 6. Relative quantitative values of differential metabolites in tilapia skins during the steaming process.

No.	Class	compound name	CAS	rt	mz	0 min	10 min	30 min	60 min
1	Organic oxygen compounds (8 differential metabolites)	aldehydo-d-xylose		156.14	149.04	0.18446±0.04105	0.01228±0.00565↓	0.01212±0.00122	0.00812±0.00240
2		2,3-dihydroxybutanedioic acid	87-69-4	29.34	149.01	0.07066±0.01315	0.04788±0.01890	0.06200±0.00940	0.03186±0.00285↓
3		(2r)-2-hydroxy-2-methylbutanenitrile		323.76	100.08	0.01511±0.00224	0.00498±0.00063↓	0.00537±0.00045	0.00525±0.00071
4		isokobusone	24173-72-6	54.67	221.15	0.25007±0.04170	0.13066±0.00762↓	0.11096±0.01047	0.09647±0.01106
5		diacetone alcohol	123-42-2	64.71	115.08	1.10154±0.11730	0.46194±0.02381↓	0.44285±0.08106	0.42449±0.01974
6		epidermin	126050-09-7	406.24	262.13	0.00090±0.00024	0.00024±0.00020↓	0.00840±0.01423	0.00012±0.00008
7		galactinol		383.63	341.11	0.00867±0.00220	0.00253±0.00035↓	0.00278±0.00152	0.00209±0.00055
8		4-oxo-2-nonenal		37.92	155.11	0.03219±0.00357	0.05483±0.00409↑	0.06204±0.02031	0.03739±0.01320
9	Organic acids and derivatives (48 differential metabolites)	pyruvic acid	127-17-3	104.43	87.01	2.44827±0.14836	1.02662±0.43393↓	1.42895±0.39559	1.10986±0.35123
10		ketoleucine	816-66-0	37.29	129.06	0.7828±0.16076	0.24900±0.07622↓	0.18720±0.05936	0.12847±0.01593
11		2-ketobutyric acid	600-18-0	243.57	101.02	0.17019±0.02169	0.08187±0.01243↓	0.07390±0.00452	0.07368±0.00804
12		l-norleucine	327-57-1	287.23	130.09	0.28869±0.03193	0.03409±0.00911↓	0.03108±0.01394	0.03394±0.00700
13		l-valine	72-18-4	310.37	118.09	0.51933±0.10378	0.24784±0.03134↓	0.23390±0.02432	0.25731±0.02686
14		sarcosine	107-97-1	371.57	88.04	0.32605±0.08685	0.06112±0.01530↓	0.0918±0.00463	0.05154±0.00381
15		5-aminopentanoic acid	660-88-8	412.26	118.09	0.01968±0.00301	0.01210±0.00108↓	0.01279±0.00063	0.01221±0.00133
16		creatine	57-00-1	387.96	132.08	1.38906±0.24653	0.18139±0.13582↓	0.25171±0.09021	0.17724±0.15702
17		prolyl-histidine		321.27	253.13	0.00189±0.00057	0.00032±0.00022↓	0.00021±0.00014	0.00024±0.00018
18		taurine	107-35-7	314.49	124.01	0.44540±0.11442	0.03007±0.01186↓	0.03180±0.01547	0.02930±0.01355
19		proline betaine	471-87-4	292.64	144.10	0.04893±0.01136	0.01001±0.00185↓	0.01064±0.00312	0.02025±0.00824
20		3,3,5-triiodo-l-thyronine-beta-d-glucuronoside	328-39-2	287.99	132.10	0.06010±0.00874	0.01663±0.00903↓	0.02337±0.00630	0.02125±0.00070
21		d-proline	344-25-2	332.36	116.07	0.44222±0.04289	0.05943±0.03599↓	0.08303±0.05987	0.09988±0.02306
22		d-alanine	338-69-2	371.12	90.06	0.10730±0.01759	0.02277±0.00633↓	0.02395±0.00352	0.01976±0.00663
23		malonic acid	141-82-2	388.36	103.00	0.57172±0.07371	0.24428±0.01159↓	0.25721±0.02077	0.16164±0.05763
24		acetylglycine	543-24-8	639.51	116.03	0.03956±0.00403	0.02125±0.00158↓	0.01790±0.00096↓	0.01588±0.00060↓
25		l-trans-4-methyl-2-pyrrolidinecarboxylic acid	23009-50-9	328.91	130.09	0.02453±0.00335	0.00355±0.00065↓	0.00508±0.00134	0.00525±0.00156
26		n-acetylhistidine	39145-52-3	322.72	198.09	0.08410±0.03550	0.02052±0.01220↓	0.01220±0.00426	0.01209±0.00936
27		hydroxypyruvic acid	1113-60-6	559.55	103.00	0.03780±0.00152	0.01924±0.00080↓	0.01720±0.00185	0.01387±0.00192

28	3-hydroxycapric acid	14292-26-3	95.70	187.13	0.09869±0.02778	0.04667±0.01833	0.04821±0.00374	0.02879±0.00323↓
29	2-hydroxybutyric acid	600-15-7	188.07	103.04	0.14533±0.05129	0.03312±0.01404↓	0.02417±0.00983	0.01765±0.00918
30	glycolic acid	79-14-1	466.36	147.00	0.05682±0.00212	0.06322±0.00330↑	0.07547±0.00802	0.06095±0.03461
31	guanidoacetic acid	352-97-6	377.04	118.06	0.00978±0.00238	0.00457±0.00046↓	0.00478±0.00054	0.00476±0.00071
32	pyroglutamic acid	98-79-3	303.73	128.03	1.25069±0.15389	0.31686±0.05614↓	0.30533±0.10195	0.27446±0.08258
33	l-phenylalanine	63-91-2	281.21	164.07	0.15977±0.02700	0.01459±0.00714↓	0.01397±0.00386	0.01175±0.00214
34	3-sulfinato-l-alaninate		139.98	152.00	0.16926±0.04541	0.03339±0.02528↓	0.03136±0.01462	0.01470±0.01049
35	n-acetyl-l-methionine	65-82-7	204.15	190.05	0.05623±0.02146	0.01102±0.00670↓	0.00641±0.00289	0.00511±0.00149
36	dimethylmalonic acid	595-46-0	55.65	131.03	0.03103±0.00259	0.01440±0.00146↓	0.01105±0.00107↓	0.00985±0.00106
37	prolyl-threonine		344.42	217.12	0.00206±0.00027	0.00051±0.00006↓	0.00048±0.00013	0.00052±0.00008
38	urea	57-13-6	611.03	171.00	0.13550±0.01748	0.02113±0.00716↓	0.03217±0.01150	0.03565±0.01807
39	l-proline	147-85-3	332.70	114.06	0.13137±0.01344	0.01167±0.00510↓	0.01715±0.00568	0.01586±0.00252
40	l-cysteine	52-90-4	140.12	178.02	0.02090±0.00292	0.00444±0.00100↓	0.00417±0.00027	0.00356±0.00034
41	3-sulfinoalanine	1115-65-7	161.31	152.00	0.04361±0.00953	0.02067±0.00247↓	0.01594±0.00143↓	0.01551±0.00158
42	n-a-acetyl-l-arginine	155-84-0	395.22	217.13	0.01394±0.00314	0.00239±0.00177↓	0.00189±0.00073	0.00188±0.00075
43	arginyl-alanine		451.93	246.16	0.00537±0.00052	0.00381±0.00047↓	0.00453±0.00073	0.00500±0.00041
44	l-3-cyanoalanine		212.71	113.03	0.04650±0.00769	0.02465±0.00538↓	0.02018±0.00099	0.02992±0.01856
45	succinic acid	110-15-6	666.44	147.00	0.05081±0.00473	0.06929±0.01380	0.08672±0.01863	0.12160±0.00966↑
46	n-acetylleucine	1188-21-2	248.25	172.10	0.03386±0.00212	0.01843±0.00305↓	0.01587±0.00156	0.01532±0.00471
47	citrulline 2	372-75-8	987.08	71.00	0.15445±0.00355	0.17699±0.01696	0.17732±0.01198	0.19903±0.00363↑
48	indoxyl sulfate	487-94-5	25.85	212.00	0.03610±0.02107	0.01589±0.00965	0.01104±0.00317	0.00382±0.00254↓
49	prolyl-valine		304.59	215.14	0.00163±0.00020	0.00104±0.00009↓	0.00115±0.00020	0.00132±0.00020
50	4-aminobutyric acid 3	56-12-2	537.37	141.00	0.17553±0.00287	0.18936±0.00420↑	0.15924±0.02740	0.18551±0.01565
51	methionine 2	63-68-3	842.60	244.00	0.03717±0.00105	0.02449±0.00225↓	0.01514±0.00980	0.01382±0.01203
52	creatinine	60-27-5	181.4	112.1	0.09360±0.00921	0.09267±0.02204	0.21311±0.06920↑	0.34103±0.17656
53	3-hydroxypropionic acid 1	503-66-2	521.34	73.00	13.20578±1.03774	11.32453±0.52861↓	9.10112±2.28401	11.70233±1.24457
54	n-(5-methyl-3-oxohexyl)alanine		595.39	202.14	0.00247±0.00024	0.00286±0.00033	0.00222±0.00021↓	0.00241±0.00006
55	parabanic acid	120-89-8	714.05	113.00	0.20822±0.01069	0.09072±0.00454↓	0.11984±0.05145	0.08648±0.01714
56	2-hydroxyethanesulfonate	107-36-8	118.49	124.99	0.65166±0.16865	0.16366±0.07626↓	0.11587±0.04083	0.09251±0.02438

57	Lipids and lipid-like molecules (127 differential metabolites)	16-hydroxy hexadecanoic acid		9.19	271.23	0.00262±0.00048	0.00087±0.00037↓	0.00090±0.00015	0.00103±0.00020
58		isopalmitic acid	25354-97-6	40.67	255.23	36.05156±4.86945	15.72490±1.04967↓	18.05257±1.62955	16.97073±0.97633
59		2-hydroxystearic acid	629-22-1	51.15	299.26	1.58860±0.17586	0.57241±0.21760↓	0.56386±0.06578	0.52351±0.09285
60		tridecanoic acid	638-53-9	41.25	213.19	0.12782±0.01313	0.04897±0.02370↓	0.04924±0.01718	0.04558±0.02195
61		alpha-dimorphecolic acid	73543-67-6	24.21	297.24	0.00251±0.00027	0.00100±0.00022↓	0.00116±0.00034	0.00124±0.00041
62		methylsuccinic acid	498-21-5	377.52	131.03	0.12154±0.00071	0.06258±0.00821↓	0.08705±0.05315	0.04893±0.00284
63		eicosapentaenoic acid	10417-94-4	43.98	301.22	1.02264±0.17334	0.29312±0.07860↓	0.36227±0.01696	0.38634±0.08295
64		9,10-epoxyoctadecenoic acid		37.28	295.23	3.15902±0.51045	0.73772±0.18293↓	0.70759±0.10697	0.73015±0.08219
65		valeric acid	109-52-4	53.57	101.06	0.24927±0.02497	0.12882±0.01579↓	0.11391±0.00759	0.13073±0.05512
66		oleamide	301-02-0	93.25	282.28	0.01171±0.00339	0.00684±0.00142	0.00713±0.00168	0.01428±0.00332↑
67		5-kete	126432-17-5	46.35	317.21	0.36119±0.04312	0.04597±0.02268↓	0.07656±0.01047	0.10927±0.02764
68		docosahexaenoic acid	6217-54-5	41.23	327.23	10.10619±1.29171	2.88931±0.70122↓	4.00948±0.15875	4.66235±0.84253
69		8-isoprostaglandin f2a	27415-26-5	177.24	353.23	0.03954±0.01085	0.00783±0.00407↓	0.01116±0.00396	0.00800±0.00034
70		succinic acid semialdehyde	692-29-5	184.65	101.02	0.52249±0.12718	0.25558±0.09809↓	0.16649±0.01835	0.15615±0.00719
71		dodecanoic acid	143-07-7	37.35	199.17	0.55183±0.14694	0.20126±0.04508↓	0.14882±0.02657	0.14623±0.03964
72		stearidonic acid	20290-75-9	40.69	275.20	0.47144±0.02598	0.15541±0.01567↓	0.12781±0.04753	0.13308±0.01178
73		(10e,12z)-(9s)-9-hydroperoxyoctadeca-10,12-dienoic acid	29774-12-7	38.48	311.22	3.66817±0.25830	0.35626±0.22933↓	0.29841±0.06572	0.35907±0.07109
74		pantothenol	81-13-0	64.83	204.12	6.73646±1.28442	0.56827±0.17163↓	0.65362±0.33667	0.58859±0.19957
75		l-palmitoylcarnitine	2364-67-2	201.27	400.34	0.00422±0.00098	0.00231±0.00057↓	0.00394±0.00129	0.00567±0.00182
76		prostaglandin a2	13345-50-1	55.21	333.21	0.15588±0.02277	0.04816±0.02821↓	0.05603±0.02870	0.03200±0.01236
77		methyl jasmonate	1211-29-6	32.82	223.13	0.23163±0.06537	0.05324±0.01074↓	0.05566±0.00441	0.03755±0.00799↓
78		l-acetylcarnitine	3040-38-8	329.88	204.12	0.07541±0.02712	0.00800±0.00271↓	0.00764±0.00303	0.00514±0.00400
79		5-hete	70608-72-9	48.89	319.23	1.71421±0.30457	0.28616±0.07802↓	0.31565±0.05803	0.37467±0.10299
80		17-hdohe	90780-52-2	47.73	343.23	0.47351±0.06227	0.07106±0.01980↓	0.10005±0.01726	0.11811±0.02987
81		oleic acid	112-80-1	1265.34	129.00	1.17590±0.02397	0.64139±0.08381↓	1.14691±0.22045↑	1.47967±0.39400
82		17a-estradiol	57-91-0	31.158	255.17	0.00313±0.00098	0.00143±0.00050	0.00226±0.00066	0.00415±0.00075↑
83		leukotriene b4	71160-24-2	53.37	335.22	0.83825±0.18902	0.07479±0.01858↓	0.07065±0.04627	0.09807±0.03224
84		palmitic amide	629-54-9	33.99	256.26	0.04001±0.00329	0.06581±0.00489↑	0.07434±0.00447	0.06803±0.00718
85		palmitic acid	57-10-3	1171.21	117.00	4.61601±0.21073	4.10426±0.51042	5.07322±0.24105↑	6.16643±0.75478

86	13-l-hydroperoxylinoleic acid	33964-75-9	48.36	295.23	0.03692±0.00670	0.01026±0.00139↓	0.01629±0.00372	0.01884±0.00275
87	stearic acid	57-11-4	1279.07	117.00	3.12691±0.12636	3.04487±0.44028	3.58002±0.16224	4.69310±0.25403↑
88	4,8 dimethylnonanoyl carnitine		57.73	330.26	0.00919±0.00182	0.00256±0.00009↓	0.00349±0.00158	0.00381±0.00133
89	arachidonic acid	506-32-1	1342.76	67.00	0.38135±0.02420	0.19636±0.03193↓	0.30629±0.05344↑	0.44322±0.12661
90	myristoleic acid	544-64-9	41.28	225.19	0.20095±0.02275	0.07601±0.03116↓	0.07827±0.01534	0.07006±0.02725
91	13-oxoode		47.50	293.21	1.83101±0.33069	0.33048±0.04896↓	0.32935±0.21081	0.45917±0.29660
92	palmitoleic acid	373-49-9	1158.64	117.00	0.08857±0.00676	0.05257±0.00517↓	0.08459±0.01932	0.10472±0.03292
93	8,9-dihetre	192461-96-4	48.73	337.24	0.23725±0.03528	0.05873±0.00404↓	0.08439±0.01731	0.08346±0.02367
94	6-angeloylfuranofukinol		36.78	333.20	0.01127±0.00478	0.00411±0.00152	0.00485±0.00027	0.00648±0.00088↑
95	propionylcarnitine	17298-37-2	304.60	218.14	0.00379±0.00114	0.00047±0.00008↓	0.00050±0.00024	0.00035±0.00012
96	2-o-(5,8,11,14,17-eicosapentaenoyl)-1-o-hexadecylglycero-3-phosphocholine	132196-28-2	115.98	766.57	0.17043±0.12634	0.13860±0.12202	0.06437±0.01203	0.09306±0.01153↑
97	panaquinquecol 1	133921-57-0	48.63	293.21	0.01632±0.00411	0.00322±0.00044↓	0.00516±0.00088↑	0.00560±0.00074
98	2-methylglutaric acid	617-62-9	225.66	145.05	0.14942±0.03265	0.03785±0.02016↓	0.02808±0.00809	0.02262±0.00526
99	ricinoleic acid	141-22-0	37.30	297.24	1.96688±0.21434	0.74360±0.19111↓	0.58521±0.17330	0.62253±0.06505
100	azelaic acid	123-99-9	338.76	187.10	0.03664±0.00767	0.02024±0.00192↓	0.01808±0.00059	0.01839±0.00090
101	jasmonic acid	59366-47-1	35.07	209.12	0.32189±0.06333	0.05385±0.01929↓	0.04863±0.01153	0.04665±0.00888
102	5,6-dhet	213382-49-1	50.92	339.25	0.00705±0.00104	0.00298±0.00088↓	0.00895±0.00515	0.00700±0.00122
103	ethyl dodecanoate	106-33-2	680.54	227.20	0.01258±0.00131	0.00669±0.00187↓	0.00477±0.00017	0.00456±0.00060
104	3-methyl-5-pentyl-2-furanundecanoic acid	57818-37-8	219.98	337.27	0.00751±0.00040	0.00184±0.00008↓	0.00276±0.00086	0.00465±0.00134
105	2-hydroxy-3-methylbutyric acid	4026-18-0	143.59	117.06	0.71058±0.14138	0.13439±0.08220↓	0.09219±0.04123	0.06857±0.03003
106	adipic acid	124-04-9	65.17	145.05	0.08187±0.01098	0.03810±0.01053↓	0.03231±0.00893	0.03109±0.00329
107	citraconic acid	498-23-7	65.06	129.02	0.26092±0.07684	0.02651±0.00404↓	0.02855±0.00468	0.01511±0.01057
108	pentadecanoic acid	1002-84-2	1112.69	117.00	0.02218±0.00226	0.01589±0.00254↓	0.02389±0.00455	0.03043±0.00416
109	citramalic acid	2306-22-1	65.93	147.03	0.11072±0.01138	0.02523±0.00613↓	0.01748±0.00582	0.01129±0.00231
110	l-malic acid	97-67-6	248.25	133.01	1.05847±0.05831	0.41419±0.07077↓	0.46709±0.11444	0.45331±0.01337
111	d-malic acid	636-61-3	262.38	133.01	0.19357±0.01166	0.07237±0.01821↓	0.08434±0.02499	0.07569±0.00696
112	citraconic acid 4	498-23-7	696.37	73.00	0.05517±0.00575	0.06573±0.00233↑	0.06682±0.01080	0.08836±0.01178
113	resolvin d2	82864-77-5	56.49	359.22	0.00442±0.00158	0.00099±0.00029↓	0.00200±0.00039↑	0.00256±0.00031
114	dihydrolipoate	462-20-4	66.25	207.05	0.05500±0.00933	0.00934±0.00078↓	0.00721±0.00056↓	0.00703±0.00267

115	cholic acid	81-25-4	233.77	407.28	10.51393±2.38824	3.06002±3.11327↓	0.81136±0.03192	1.05639±0.70733
116	tetradecanoylcarnitine	25597-07-3	205.32	372.31	0.00180±0.00044	0.00060±0.00027↓	0.00111±0.00043	0.00186±0.00124
117	chenodeoxycholic acid	474-25-9	149.34	391.29	0.07012±0.01829	0.01604±0.00335↓	0.01195±0.00443	0.00584±0.00111
118	nandrolone	434-22-0	36.28	275.20	0.01394±0.00265	0.00807±0.00097↓	0.00888±0.00129	0.01032±0.00215
119	3,5,9-trihydroxyergost-7-en-6-one	211486-13-4	31.20	447.35	0.01179±0.00085	0.00555±0.00347↓	0.00182±0.00018	0.00278±0.00199
120	18-oxocortisol	2410-60-8	25.26	377.20	0.00166±0.00017	0.00065±0.00012↓	0.00073±0.00030	0.00088±0.00023
121	butyrylcarnitine	25576-40-3	283.05	232.15	0.00444±0.00143	0.00045±0.00018↓	0.00052±0.00015	0.00047±0.00017
122	pivaloylcarnitine	98299-38-8	264.38	246.17	0.00341±0.00055	0.00026±0.00013↓	0.00052±0.00050	0.00045±0.00036
123	3-beta-hydroxy-4-beta-methyl-5-alpha-cholest-7-ene-4-alpha-carbaldehyde		31.08	429.37	0.04103±0.00589	0.02488±0.00804↓	0.02955±0.00604	0.03953±0.01800
124	tsugaric acid b	201045-20-7	31.08	529.39	0.00247±0.00006	0.00130±0.00032↓	0.00136±0.00033	0.00137±0.00002
125	androsterone 1	53-41-8	1432.87	91.00	0.06203±0.01356	0.03180±0.00885↓	0.04862±0.00648	0.06891±0.01652
126	20-hydroxy-pge2	57930-95-7	57.25	367.21	0.04181±0.03061	0.00463±0.00136	0.00847±0.00122↑	0.01261±0.00240
127	etonogestrel	54048-10-1	31.21	325.22	0.00467±0.00182	0.00136±0.00014	0.00256±0.00013↑	0.00339±0.00185
128	5a-cholesta-8,24-dien-3-one		32.22	383.33	0.00576±0.00304	0.01688±0.01765	0.00930±0.00129	0.01220±0.00100↑
129	lysopc(16:1(9z)/0:0)	76790-27-7	222.25	494.32	0.04674±0.00552	0.03534±0.01382	0.06676±0.00507↑	0.10641±0.03889
130	cortisone	53-06-5	25.84	421.20	0.04875±0.01542	0.02321±0.00356↓	0.02236±0.00505	0.02348±0.01056
131	lysope(0:0/20:4(5z,8z,11z,14z))		216.51	502.29	0.08431±0.01087	0.02046±0.00395↓	0.03478±0.01197	0.06802±0.01093↑
132	lysope(16:0/0:0)		227.14	454.29	0.04480±0.00497	0.02353±0.00370↓	0.05039±0.00456↑	0.08727±0.03229
133	lysope(22:6(4z,7z,10z,13z,16z,19z)/0:0)		222.20	526.29	0.01251±0.00791	0.01919±0.00249	0.05664±0.01413↑	0.11859±0.02461↑
134	lysope(20:4(8z,11z,14z,17z)/0:0)		184.18	502.29	0.00359±0.00065	0.00097±0.00016↓	0.00101±0.00046	0.00108±0.00031
135	pc(20:3(8z,11z,14z)/20:1(11z))		161.54	838.63	0.04104±0.00890	0.04200±0.00315	0.05453±0.00333↑	0.05771±0.01109
136	pc(22:5(4z,7z,10z,13z,16z)/20:5(5z,8z,11z,14z,17z))		103.75	854.57	0.00983±0.01080	0.00346±0.00043	0.00385±0.00080	0.00570±0.00031↑
137	lysope(18:1(9z)/0:0)	89576-29-4	223.64	478.29	0.14293±0.02321	0.06042±0.01313↓	0.10560±0.01249↑	0.18100±0.04112↑
138	lysope(22:5(4z,7z,10z,13z,16z)/0:0)		220.42	528.31	0.02845±0.00119	0.04603±0.00867	0.09839±0.02087↑	0.18544±0.05483
139	lysopa(18:1(9z)/0:0)		217.95	435.25	0.01641±0.00147	0.00802±0.00229↓	0.01146±0.00129	0.01408±0.00355
140	pc(20:3(8z,11z,14z)/p-18:0)		162.68	796.62	0.15102±0.01833	0.10972±0.01751↓	0.11118±0.02162	0.16378±0.02652
141	lysopa(16:0/0:0)		219.76	409.24	0.02928±0.00367	0.00963±0.00267↓	0.01382±0.00246	0.01922±0.00545
142	lysopc(14:0/0:0)	20559-16-4	225.82	468.31	0.04225±0.01381	0.02106±0.00468	0.04250±0.00460↑	0.03579±0.03264

143	lysopc(p-16:0)		212.45	480.34	0.25436±0.02797	0.13483±0.06527↓	0.16530±0.07395	0.21961±0.07551
144	lysopc(15:0)		219.25	482.32	0.01959±0.00475	0.01629±0.00136	0.03956±0.00343↑	0.04932±0.01623
145	pe(p-18:1(11z)/18:3(6z,9z,12z))		31.88	724.53	0.00247±0.00021	0.00062±0.00032↓	0.00056±0.00029	0.00175±0.00174
146	pc(18:1(9z)/p-18:1(11z))		165.50	770.60	0.13615±0.01898	0.10213±0.00748↓	0.10154±0.05394	0.13280±0.02547
147	lysopc(20:0/0:0)	108341-80-6	213.66	552.40	0.01049±0.00240	0.00589±0.00134↓	0.01255±0.00387↑	0.01933±0.00639
148	lysope(22:4(7z,10z,13z,16z)/0:0)		152.37	530.32	0.00225±0.00041	0.00078±0.00023↓	0.00082±0.00004	0.00045±0.00018↓
149	lysopc(18:1(9z))	3542-29-8	218.16	522.35	0.34404±0.04302	0.32587±0.08032	0.59020±0.08230↑	0.90498±0.31777
150	2-acetyl-1-alkyl-sn-glycero-3-phosphocholine	74389-68-7	217.62	524.37	0.43632±0.04674	0.22430±0.07950↓	0.37057±0.09440	0.65061±0.26522
151	pc(18:3(6z,9z,12z)/18:0)		166.05	784.58	0.98275±0.05250	0.76314±0.10142↓	0.93150±0.08466	1.14634±0.27979
152	pc(22:5(4z,7z,10z,13z,16z)/p-18:0)		159.24	820.62	0.15215±0.02442	0.10088±0.01074↓	0.12048±0.01194	0.14218±0.01144
153	pc(22:5(7z,10z,13z,16z,19z)/16:1(9z))		162.70	806.57	0.46840±0.03208	0.32796±0.02317↓	0.40410±0.06937	0.53057±0.10050
154	pc(22:5(7z,10z,13z,16z,19z)/20:4(5z,8z,11z,14z))		156.95	856.58	0.06317±0.00491	0.03772±0.00845↓	0.04255±0.01028	0.05432±0.00465
155	pc(18:2(9z,12z)/18:0)		137.37	786.60	0.15764±0.01260	0.20042±0.02228↑	0.21415±0.05609	0.53975±0.41283
156	pe(16:0/18:2(9z,12z))		170.97	714.51	0.33503±0.02594	0.15727±0.06622↓	0.13291±0.03827	0.16226±0.06431
157	pc(22:6(4z,7z,10z,13z,16z,19z)/20:1(11z))		159.21	860.62	0.01844±0.00257	0.01244±0.00170↓	0.01542±0.00191	0.01738±0.00106
158	pc(p-16:0/16:0)		418.13	718.57	0.03717±0.00105	0.00006±0.00006↓	0.00839±0.01423	0.00068±0.00060
159	pc(20:2(11z,14z)/15:0)		109.67	772.58	0.01115±0.00327	0.00845±0.00173	0.01585±0.00263↑	0.01551±0.00378
160	pc(22:4(7z,10z,13z,16z)/p-18:0)		160.35	822.63	0.04945±0.00922	0.03631±0.00408	0.04501±0.00216↑	0.05516±0.00596
161	ps(20:5(5z,8z,11z,14z,17z)/20:0)		209.14	838.56	0.01125±0.00131	0.02461±0.00767↑	0.02027±0.00140	0.02061±0.00524
162	pe(22:4(7z,10z,13z,16z)/14:0)		31.71	740.53	0.00162±0.00025	0.00045±0.00047↓	0.00865±0.01402	0.00107±0.00076
163	lysopc(20:4(8z,11z,14z,17z))		216.98	544.34	0.11095±0.01560	0.14319±0.05175	0.23351±0.03788	0.43649±0.03969↑
164	lysopc(22:5(4z,7z,10z,13z,16z))		214.13	570.35	0.06554±0.01246	0.06155±0.01103	0.11470±0.02359↑	0.20133±0.04701↑
165	lysopc(22:6(4z,7z,10z,13z,16z,19z))		215.85	568.34	0.03448±0.00278	0.03423±0.01355	0.06501±0.01099↑	0.11100±0.02169↑
166	lysopc(18:3(6z,9z,12z))		175.16	518.32	0.00208±0.00022	0.00045±0.00008↓	0.00084±0.00048	0.00088±0.00026
167	lysosm(d18:1)	1670-26-4	269.48	465.34	0.01207±0.02075	0.00007±0.00001	0.00049±0.00008↑	0.00129±0.00032↑
168	lysopc(18:2(9z,12z))		219.98	520.34	0.24392±0.03115	0.23280±0.03893	0.37320±0.07561↑	0.69600±0.28553
169	linoleic acid	60-33-3	1262.14	67.00	1.11022±0.03634	0.56606±0.07280↓	0.96255±0.22074↑	1.33058±0.40563
170	dihydrojasmonic acid	98674-52-3	38.35	211.13	0.29391±0.03432	0.01519±0.00319↓	0.01377±0.01076	0.01429±0.00591
171	(9s,10e,12z,15z)-9-hydroxy-10,12,15-octadecatrienoic acid	89886-42-0	36.81	277.22	0.08449±0.00907	0.04308±0.00759↓	0.04637±0.00486	0.05258±0.00859

172		sm(d18:1/20:0)		606.03	759.63	0.03717±0.00105	0.00050±0.00020↓	0.00044±0.00046	0.00113±0.00066
173		pc(22:5(7z,10z,13z,16z,19z)/15:0)		176.30	794.57	0.02555±0.00657	0.01602±0.00040	0.02205±0.00059↑	0.01972±0.00504
174		sm(d18:0/18:1(9z))		203.40	731.60	0.02387±0.00298	0.04512±0.00677↑	0.02987±0.00065	0.02490±0.00242↓
175		6-o-acetylaustroinulin	75207-46-4	194.41	365.28	0.00956±0.00270	0.00082±0.00003↓	0.00105±0.00059	0.00150±0.00063
176		persicaxanthin	80952-82-5	160.41	385.27	0.00648±0.00084	0.00430±0.00089↓	0.00609±0.00150	0.00615±0.00183
177		volemolide	125974-96-1	38.04	347.26	0.02142±0.00351	0.01000±0.00074↓	0.01574±0.00325↑	0.01633±0.00364
178		alpha-tocopherol succinate	4345-03-3	31.08	531.41	0.00774±0.00082	0.00506±0.00071↓	0.00395±0.00067	0.00414±0.00048
179		dg(18:4(6z,9z,12z,15z)/18:2(9z,12z)/0:0)		31.21	613.48	0.01046±0.00105	0.00661±0.00156↓	0.00786±0.00179	0.00848±0.00153
180		dg(22:6(4z,7z,10z,13z,16z,19z)/16:0/0:0)		31.14	641.51	0.00526±0.00013	0.00300±0.00059↓	0.00350±0.00044	0.00402±0.00098
181		dg(20:3(8z,11z,14z)/18:2(9z,12z)/0:0)		31.07	643.53	0.00637±0.00054	0.00376±0.00097↓	0.00379±0.00120	0.00440±0.00092
182		dg(20:5(5z,8z,11z,14z,17z)/20:2(11z,14z)/0:0)		31.10	667.53	0.00673±0.00026	0.00385±0.00073↓	0.00402±0.00110	0.00446±0.00081
183		pc(22:6(4z,7z,10z,13z,16z,19z)/15:0)		176.30	792.55	0.01221±0.00387	0.00915±0.00162	0.01320±0.00048↑	0.01230±0.00190
184	Organohetero-cyclic compounds (27 differential metabolites)	adenine	73-24-5	167.56	134.05	0.27509±0.06115	0.08657±0.01454↓	0.11306±0.01960	0.15724±0.00573↑
185		3-methyladenine	5142-23-4	262.02	150.08	0.07075±0.03249	0.01414±0.00662↓	0.01104±0.00368	0.01032±0.00547
186		hypoxanthine	68-94-0	177.81	135.03	82.92200±15.91431	26.56505±4.86084↓	25.20943±9.07874	20.94973±4.98141
187		1-methylguanine	938-85-2	205.48	164.06	0.71546±0.10583	0.29218±0.04980↓	0.28924±0.09133	0.22489±0.01256
188		guanine 1	73-40-5	1214.06	352.00	2.97227±0.04421	2.42828±0.21519↓	3.33762±1.22214	3.65478±0.08315
189		2,8-dihydroxyadenine	30377-37-8	252.53	168.05	0.05706±0.00327	0.04962±0.00187↓	0.05864±0.01362	0.06233±0.00562
190		niacinamide	98-92-0	58.83	123.06	0.10385±0.01715	0.02779±0.00460↓	0.03431±0.00608	0.03474±0.00649
191		2-hydroxypyridine	142-08-5	435.77	152.00	3.33713±0.13868	3.78753±0.10885↑	3.53041±0.16705	3.74081±0.45580
192		nicotinic acid	59-67-6	222.49	122.02	0.31135±0.02908	0.08931±0.03360↓	0.06479±0.01589	0.05878±0.01022
193		urocanic acid	104-98-3	297.32	139.05	0.40952±0.00790	0.21727±0.02215↓	0.26133±0.14109	0.18527±0.03981
194		imidazoleacetic acid	645-65-8	66.25	125.03	1.34670±0.38130	0.08746±0.04395↓	0.09596±0.04491	0.07890±0.03751
195		demethylated antipyrine	89-25-8	49.66	175.09	0.00870±0.00263	0.00159±0.00034↓	0.00164±0.00035	0.00164±0.00015
196		propylpyrazine	18138-03-9	63.43	123.09	0.01731±0.00222	0.02152±0.00134↑	0.02228±0.00507	0.02026±0.00275
197		arborinine	5489-57-6	346.87	286.10	0.00037±0.00015	0.00015±0.00013	0.00012±0.00004	0.00021±0.00004↑
198		dihydrouracil	504-07-4	650.67	113.04	0.03533±0.00574	0.01147±0.00279↓	0.01126±0.00427	0.01178±0.00014
199		n-(14-methylhexadecanoyl)pyrrolidine	260058-83-1	196.21	324.33	0.00258±0.00092	0.00027±0.00006↓	0.00010±0.00004↓	0.01929±0.01652
200		2-pyrrolidinone	616-45-5	50.68	86.06	0.02336±0.00051	0.02720±0.00072↑	0.03328±0.00307↑	0.02679±0.00050↓

201		pterin	2236-60-4	201.93	162.04	0.17718±0.05450	0.07397±0.02472↓	0.07747±0.04678	0.04432±0.03801
202		isoxanthopterin	529-69-1	1222.55	265.00	0.03717±0.00105	0.01514±0.00618↓	0.01845±0.00553	0.02095±0.00831
203		cafestol	469-83-0	61.49	317.21	0.00897±0.00267	0.00315±0.00097↓	0.00340±0.00042	0.00434±0.00047
204		(+)-2,3-dihydro-3-methyl-1h-pyrrole		399.57	84.08	0.00237±0.00049	0.00624±0.00126↑	0.00386±0.00138	0.00506±0.00110
205		indoleacetaldehyde	2591-98-2	33.88	158.06	0.11668±0.03187	0.02850±0.00844↓	0.01746±0.00723	0.01462±0.00724
206		1-pyrroline	5724-81-2	332.60	70.06	0.00231±0.00027	0.00144±0.00009↓	0.00158±0.00010	0.00149±0.00014
207		tetrahydroneopterin	25976-00-5	402.18	258.12	0.00185±0.00024	0.00224±0.00007	0.00266±0.00020↑	0.00189±0.00004↓
208		7-aminomethyl-7-carbaguanine		145.46	180.09	0.03305±0.00861	0.00737±0.00202↓	0.00765±0.00240	0.00715±0.00254
209		tetrahydro-2-methyl-2-thiophenethiol	62308-60-5	247.97	135.03	0.05691±0.00204	0.04238±0.00574↓	0.05743±0.01488	0.05674±0.00206
210		biotin sulfone	40720-05-6	325.20	277.08	0.00067±0.00011	0.00068±0.00016	0.00101±0.00010↑	0.00120±0.00007
211	Organic nitrogen compounds (4 differential metabolites)	choline	62-49-7	319.18	104.11	0.96479±0.20037	0.12759±0.02417↓	0.21646±0.00824↑	0.22518±0.08274
212		l-carnitine	541-15-1	379.18	162.11	0.10206±0.02886	0.01761±0.00239↓	0.02159±0.00550	0.02156±0.00887
213		beta-guanidinopropionic acid		371.85	132.08	6.32723±1.16547	1.06133±0.36716↓	1.06112±0.43372	0.86336±0.46536
214		trimethylamine n-oxide	1184-78-7	351.72	76.08	0.01517±0.00631	0.00358±0.00101↓	0.00416±0.00113	0.00302±0.00065
215	Homogeneous non-metal compounds (2 differential metabolites)	hydrogen phosphate	14066-19-4	400.69	96.96	0.09520±0.02091	0.03542±0.01925↓	0.01454±0.01711	0.03603±0.00752
216		phosphate	7664-38-2	630.37	299.00	5.00146±0.73822	7.60873±0.92523↑	9.33461±1.35655	13.21157±1.42310↑
217	Benzenoids (15 differential metabolites)	benzoic acid	65-85-0	36.21	121.03	0.74234±0.24196	0.21179±0.05435↓	0.16060±0.01397	0.16108±0.00734
218		4-dodecylbenzenesulfonic acid		691.16	325.18	0.02707±0.00098	0.01780±0.00274↓	0.01298±0.00157	0.01375±0.00497
219		terephthalic acid	100-21-0	348.91	165.02	0.73363±0.06305	0.39408±0.04163↓	0.32365±0.01040↓	0.30582±0.01564
220		2-hydroxy-6-(8,11,14-pentadecatrienyl)benzoic acid	103904-73-0	35.85	343.23	0.01792±0.00269	0.00813±0.00168↓	0.01054±0.00310	0.01182±0.00341
221		phthalic acid	88-99-3	617.91	165.02	0.01560±0.00111	0.00775±0.00205↓	0.00680±0.00056↓	0.00958±0.00254
222		antibiotic sb 202742	83173-24-4	184.23	371.26	0.00908±0.00261	0.00313±0.00203↓	0.00140±0.00058	0.00214±0.00070
223		hexylresorcinol	136-77-6	36.78	195.14	0.01090±0.00145	0.00607±0.00064↓	0.00680±0.00087	0.00548±0.00399
224		vanillin	121-33-5	44.79	151.05	0.03027±0.01540	0.00360±0.00345↓	0.00970±0.00039	0.00648±0.00109↓
225		p-mentha-1,3,5,8-tetraene	1195-32-0	33.48	133.10	0.03484±0.00380	0.02575±0.00311↓	0.02908±0.00247	0.02958±0.00253
226		alpha-methylstyrene		35.67	119.09	0.02966±0.00401	0.02209±0.00193↓	0.02581±0.00394	0.02367±0.00390
227		diflubenzuron	35367-38-5	178.94	309.03	0.08159±0.02278	0.02779±0.00714↓	0.02375±0.01121	0.01523±0.00658
228		(s)-mandelic acid	17199-29-0	787.12	71.00	0.25358±0.01715	0.08977±0.00993↓	0.09258±0.00737	0.09790±0.00816

229		aniline	62-53-3	37.43	94.06	0.00199±0.00024	0.00303±0.00050↑	0.00263±0.00018	0.00243±0.00060
230		gingerol	58253-27-3	54.65	293.18	0.58214±0.11193	0.34732±0.04448↓	0.28702±0.02131	0.22501±0.03526
231		5-nitro-2-propoxyaniline	553-79-7	56.76	197.08	0.00132±0.00031	0.00199±0.00026↑	0.00161±0.00022	0.00121±0.00039
232	Nucleosides, nucleotides, and analogues (8 differential metabolites)	inosine	58-63-9	232.84	267.07	0.08893±0.03618	0.01273±0.00059↓	0.01643±0.00650	0.02001±0.00348
233		adenosine	58-61-7	181.22	266.09	0.01536±0.00314	0.01610±0.00199	0.02010±0.00340	0.02890±0.00345↑
234		deoxyinosine	890-38-0	191.53	251.08	0.31275±0.04879	0.03431±0.01571↓	0.03419±0.00709	0.02912±0.00619
235		deoxyguanosine	961-07-9	250.55	266.09	0.01861±0.00360	0.00342±0.00159↓	0.00221±0.00038	0.00226±0.00070
236		cytidine	65-46-3	258.79	242.08	0.03029±0.00521	0.00461±0.00189↓	0.00534±0.00227	0.00515±0.00046
237		cytarabine	675-20-7	53.06	100.08	0.00933±0.00035	0.00110±0.00042↓	0.00141±0.00019	0.00171±0.00061
238		5'-methylthioadenosine	2457-80-9	90.32	298.10	0.00090±0.00012	0.00804±0.00250↑	0.00979±0.00444	0.00743±0.00289
239		2-deoxyuridine	951-78-0	628.07	85.00	0.04341±0.00267	0.04190±0.00648	0.04203±0.00114	0.04611±0.00141↑
240	Organooxygen compounds (6 differential metabolites)	ribitol	488-81-3	243.56	151.06	3.98901±0.25347	2.02899±0.20023↓	1.67004±0.06262↓	1.66065±0.04204
241		d-arabitol	488-82-4	98.57	151.06	0.18795±0.04235	0.01998±0.00338↓	0.02198±0.00177	0.01966±0.00314
242		ribose	24259-59-4	935.26	103.00	0.08794±0.02484	0.01743±0.00553↓	0.01555±0.00074	0.01856±0.00753
243		myo-inositol	87-89-8	1191.47	73.00	0.34732±0.10167	0.03664±0.01093↓	0.05557±0.01243	0.06393±0.02390
244		quinceoxepine	132925-10-1	35.79	179.14	0.01368±0.00098	0.00687±0.00133↓	0.00752±0.00085	0.00791±0.00096
245		acetol 4	116-09-6	958.02	57.00	0.09257±0.00278	0.09753±0.02253	0.10046±0.00618	0.11715±0.00748↑
246	Phenylpropan-oids and polyketides (2 differential metabolites)	l-3-phenyllactic acid	20312-36-1	116.58	165.06	0.07778±0.01356	0.02601±0.00813↓	0.02877±0.01532	0.01440±0.00238
247		ethyl trans-p-methoxycinnamate	24393-56-4	36.84	291.19	0.00718±0.00125	0.00342±0.00023↓	0.00420±0.00059	0.00374±0.00027
248	Organonitroge-n compounds (2 differential metabolites)	pentanenitrile	110-59-8	328.87	84.08	0.00565±0.00022	0.00322±0.00025↓	0.00383±0.00010↑	0.00353±0.00001↓
249		triethanolamine	102-71-6	176.32	150.11	0.03781±0.01083	0.00757±0.00500↓	0.00611±0.00049	0.00702±0.00295
250	Alkaloids and derivatives (3 differential metabolites)	homoarecoline	28125-84-0	34.50	170.12	0.02269±0.00439	0.04363±0.00952↑	0.06774±0.04664	0.04370±0.01107
251		arecaidine	499-04-7	59.20	128.08	0.03467±0.00314	0.03680±0.00502	0.03565±0.00086	0.03392±0.00061↓
252		xanthine	69-89-6	222.10	151.03	5.18385±0.42452	0.73082±0.30921↓	0.63745±0.14916	0.41097±0.12317
253	Others (33 differential metabolites)	elaidic acid	112-79-8	1268.48	129.00	0.15149±0.01199	0.09036±0.01337↓	0.17198±0.02164↑	0.21102±0.04241
254		1-methyl-1,3-cyclohexadiene	1489-56-1	35.63	95.09	0.07219±0.00788	0.05341±0.00587↓	0.07195±0.01646	0.07250±0.00455
255		3-hydroxybutyric acid	306-31-0	269.40	103.04	0.01196±0.00233	0.00526±0.00015↓	0.00479±0.00064	0.00407±0.00133

256	alanine 1	56-41-7	488.86	116.00	0.35864±0.05218	0.07219±0.01504↓	0.12242±0.04796	0.11331±0.06221
257	maleimide	541-59-3	484.22	154.00	0.01613±0.00031	0.10744±0.01419↑	0.12091±0.03633	0.13363±0.02286
258	1-monopalmitin	542-44-9	1444.98	73.00	0.07416±0.00183	0.11884±0.02251	0.19712±0.02029↑	0.28235±0.09842
259	d-talose 1	2595-98-4	1076.85	73.00	0.21734±0.07245	0.05488±0.02230↓	0.17250±0.16217	0.07084±0.00688
260	oxoproline	98-79-3	828.46	156.00	0.16018±0.01183	0.10071±0.02247↓	0.12075±0.02165	0.12360±0.05482
261	proline	34363-28-5	992.18	73.00	0.10441±0.00651	0.05289±0.01451↓	0.09233±0.01581↑	0.11639±0.02694
262	heptadecanoic acid	506-12-7	1225.51	117.00	0.03613±0.00096	0.02865±0.00504	0.04343±0.00726↑	0.05762±0.00528
263	2-monopalmitin	23470-00-0	1429.51	129.00	0.11552±0.00080	0.02449±0.00225↓	0.02356±0.00424	0.03225±0.00401
264	12-hpete		37.92	319.23	0.03215±0.00720	0.01078±0.00200↓	0.01182±0.00141	0.01335±0.00478
265	2-monoolein	3443-84-3	1503.59	67.00	0.05194±0.01021	0.02449±0.00225↓	0.02604±0.00112	0.01954±0.01609
266	valine	72-18-4	583.89	144.00	0.07759±0.00980	0.01684±0.00361↓	0.02152±0.00608	0.02030±0.00900
267	cis-gondoic acid	5561-99-9	1363.46	67.00	0.03618±0.00543	0.02288±0.00111↓	0.03608±0.00551↑	0.04842±0.00696
268	monostearin	123-94-4	1530.54	73.00	0.03152±0.00228	0.02366±0.00269↓	0.03464±0.00328↑	0.04833±0.01443
269	sulfuric acid	7664-93-9	541.50	281.00	0.11171±0.02323	0.41497±0.06771↑	0.48004±0.07400	0.33534±0.03445↓
270	abietic acid 2	514-10-3	1353.20	73.00	0.09830±0.00663	0.06634±0.01434↓	0.10970±0.00949↑	0.14898±0.04366
271	dioctyl phthalate	117-81-7	1423.48	149.00	0.01264±0.00096	0.02329±0.00309↑	0.02884±0.00715	0.02307±0.00700
272	4-hydroxypyridine	626-64-2	534.08	152.00	0.12090±0.00541	0.13008±0.00255	0.12268±0.00266↓	0.12636±0.01672
273	maleamate 1	557-24-4	858.70	73.00	0.04443±0.01281	0.06817±0.00719↑	0.06384±0.01595	0.07401±0.00762
274	4-hydroxy-6-methyl-2-pyrone	675-10-5	807.43	71.00	0.14183±0.00297	0.15033±0.02129	0.14748±0.00768	0.16687±0.00703↑
275	4-hydroxybutyrate	502-85-2	598.31	73.00	0.03246±0.00814	0.01363±0.00312↓	0.02245±0.00611	0.02824±0.00150
276	biuret 3	108-19-0	746.57	229.00	0.03717±0.00105	0.02467±0.00516↓	0.02940±0.00732	0.03359±0.00284
277	2,2-dimethylsuccinic acid	597-43-3	664.24	76.00	0.03717±0.00105	0.01882±0.01182	0.00886±0.00111	0.01583±0.00116↑
278	2,4,6-trihydroxybenzophenone	3555-86-0	1257.28	73.00	0.02390±0.01234	0.02449±0.00225	0.01601±0.00215↓	0.02938±0.00394↑
279	atrazine-2-hydroxy 5	2163-68-0	1092.80	57.00	0.05201±0.00142	0.06274±0.00123↑	0.06177±0.00651	0.06864±0.00978
280	benzoin 2	119-53-9	1063.23	149.00	0.03136±0.00184	0.00963±0.00742	0.01886±0.00187	0.03228±0.00329↑
281	allylmalonic acid	2583-25-7	689.02	171.00	0.03717±0.00105	0.02036±0.00259↓	0.02349±0.00663	0.02083±0.00071
282	tes	7365-44-8	1193.63	55.00	0.07466±0.00455	0.01772±0.00195↓	0.02336±0.00295	0.03094±0.00907
283	15-methylpalmitate		40.76	269.25	1.51213±0.05618	0.64433±0.07802↓	0.96653±0.35700	0.76600±0.06373
284	cytosine	71-30-7	224.87	112.05	0.04258±0.00979	0.01485±0.00288↓	0.01053±0.00174	0.00949±0.00183

285	5-amino-3-oxohexanoate	323.97	146.08	0.19544±0.04384	0.01564±0.00858↓	0.01891±0.00944	0.01995±0.01251

Note:
Differential metabolites in yellow means they are associated with taste.
Differential metabolites in green means they are associated with flavor.
Blue down arrows indicate the amount decrease at this time point compared with last time point.
Red up arrows indicate the amount increase at this time point compared with last time point.

Supplementary Table 7. Relative quantitative values of differential metabolites in tilapia skin soups during the steaming process.

id	Class	compound name	CAS	rt	mz	10min	30min	60min
1	Organic acids and derivatives (10 differential metabolites)	d-ornithine	348-66-3	555.30	133.10	0.01656±0.01047	0.01110±0.00305	0.00337±0.00147↓
2		n-(5-methyl-3-oxohexyl)alanine		595.39	202.14	0.00355±0.00030	0.00416±0.00013↑	0.00414±0.00064
3		4-amino-2-methylenebutanoic acid	65370-67-4	558.20	116.10	0.01042±0.00136	0.01214±0.00038↑	0.01030±0.00093↓
4		o-acetylethanolamine	1854-30-4	398.30	104.10	0.03099±0.00163	0.03690±0.00066↑	0.03514±0.00296
5		succinic acid	110-15-6	666.50	147.00	0.01070±0.00082	0.00886±0.00096	0.01380±0.00160↑
6		l-n-carboxymethylserine	17136-47-9	202.70	164.10	0.01956±0.00462	0.03889±0.00667↑	0.02351±0.02291
7		3,3,5-triiodo-l-thyronine-beta-d-glucuronoside	328-39-2	459.9	132.10	0.00496±0.00035	0.00611±0.00055↑	0.00552±0.00144
8		2-ketobutyric acid	600-18-0	71.37	101.00	0.02122±0.02699	0.15786±0.05696↑	0.24162±0.14510
9		d-lactic acid	10326-41-7	77.58	89.02	0.04042±0.01838	0.05270±0.00406	0.04306±0.00216↓
10		arginyl-alanine		452.12	246.15	0.00428±0.00006	0.00394±0.00007↓	0.00451±0.00063
11	Organic oxygen compounds (5 differential metabolites)	theaspirone a	24399-19-7	595.4	209.2	0.05715±0.00363	0.07071±0.00348↑	0.06784±0.01030
12		aminoacetone	298-08-8	595.40	74.06	0.00512±0.00123	0.00801±0.00025↑	0.00760±0.00199
13		galactinol		383.30	341.10	0.00525±0.00204	0.00583±0.00062	0.00391±0.00060↓
14		n-acetylneuraminic acid	131-48-6	373.10	308.10	0.00398±0.00171	0.00961±0.00278↑	0.01991±0.00427↑
15		tartaric acid	133-37-9	903.10	73.00	0.00669±0.00113	0.00337±0.00082↓	0.00300±0.00035
16	Lipids and lipid-like molecules (16 differential metabolites)	capric acid	334-48-5	48.03	171.10	0.44726±0.04796	0.48484±0.00681	0.45964±0.01170↓
17		butyramide	541-35-5	668.80	88.08	0.00766±0.00053	0.00941±0.00084↑	0.00827±0.00035
18		15-keto-prostaglandin e2	26441-05-4	52.60	349.20	0.00827±0.00191	0.00436±0.00149↓	0.01539±0.00994
19		(9s,10e,12z,15z)-9-hydroxy-10,12,15-octadecatrienoic acid	89886-42-0	36.81	277.22	0.00247±0.00025	0.00346±0.00007↑	0.00475±0.00077
20		prostaglandin a2	13345-50-1	53.16	333.20	0.02927±0.01441	0.07988±0.00672↑	0.07389±0.03207
21		13-l-hydroperoxylinoleic acid	33964-75-9	48.60	295.20	0.00212±0.00053	0.00275±0.00054	0.00424±0.00041↑
22		8-isoprostaglandin e1	21003-46-3	178.00	353.20	0.01637±0.00371	0.02819±0.00614↑	0.03662±0.01427
23		lysope(0:0/20:4(5z,8z,11z,14z))		222.80	502.30	0.00228±0.00063	0.00388±0.00073↑	0.00705±0.00097↑
24		lysope(18:2(9z,12z)/0:0)	85046-18-0	226.45	478.29	0.00056±0.00006	0.00061±0.00012	0.00094±0.00010↑
25		pc(16:1(9z)/p-18:1(11z))		165.59	742.57	0.00380±0.00054	0.00322±0.00026	0.00431±0.00030↑
26		ethyl abietate	631-71-0	52.12	331.26	0.00062±0.00016	0.00155±0.00052↑	0.00180±0.00054

27		lysope(22:6(4z,7z,10z,13z,16z,19z)/0:0)		222.20	526.29	0.00056±0.00009	0.00153±0.00045↑	0.00251±0.00066
28		delta-12-prostaglandin j2	87893-54-7	61.57	335.22	0.00160±0.00015	0.00185±0.00008	0.00255±0.00041↑
29		pe(p-18:1(11z)/22:5(4z,7z,10z,13z,16z))		131.48	776.56	0.00104±0.00006	0.00116±0.00003↑	0.00115±0.00014
30		pc(18:1(9z)/p-16:0)		167.9	744.6	0.00889±0.00201	0.00719±0.00065	0.01059±0.00150↑
31		lysopc(20:4(5z,8z,11z,14z))		216.80	544.30	0.00609±0.00171	0.00682±0.00161	0.01140±0.00081↑
32	Organoheterocyclic compounds (10 differential metabolites)	isoxanthopterin	529-69-1	330.30	178.00	0.04446±0.01781	0.11804±0.02648↑	0.10277±0.00265
33		2-piperidinone	675-20-7	52.14	100.10	0.08941±0.01684	0.13566±0.02105↑	0.16355±0.04064
34		indole	120-72-9	265.70	118.10	0.07330±0.00215	0.06535±0.00287↓	0.06971±0.00552
35		pyrrolidine	123-75-1	593.60	72.08	0.00409±0.00066	0.00740±0.00092↑	0.00645±0.00166
36		hypoxanthine	68-94-0	178.00	135.00	36.47409±5.72665	51.58498±6.58534↑	52.26880±11.25874
37		(+)-2,3-dihydro-3-methyl-1h-pyrrole		405.90	84.08	0.00523±0.00056	0.00677±0.00039↑	0.00432±0.00277
38		thiabendazole	148-79-8	222.00	202.00	0.02882±0.00090	0.03326±0.00172↑	0.03228±0.00537
39		1-methylpyrrolo[1,2-a]pyrazine	64608-59-9	207.30	133.10	0.01097±0.00364	0.01138±0.00337	0.02323±0.00199↑
40		pyrazine	290-37-9	25.36	81.04	0.00263±0.00073	0.00210±0.00033	0.00315±0.00048↑
41		2,5-dihydro-2,4,5-trimethyloxazole	22694-96-8	33.48	114.10	0.02741±0.00913	0.05313±0.00265↑	0.04313±0.01629
42	Others (16 differential metabolites)	inosine	58-63-9	232.10	267.10	0.02016±0.00391	0.02921±0.00247↑	0.02868±0.00937
43		7-methyladenine		120.80	150.10	0.00308±0.00141	0.00763±0.00184↑	0.01870±0.01135
44		ethyl trans-p-methoxycinnamate	24393-56-4	36.84	291.19	0.00053±0.00007	0.00071±0.00006↑	0.00063±0.00025
45		1-isopropyl-2-methylbenzene		399.50	134.10	0.00479±0.00046	0.01315±0.00111↑	0.00656±0.00254↓
46		benzoin 2	119-53-9	1063.23	149.00	0.00318±0.00058	0.00198±0.00031↑	0.00270±0.00036
47		9-phenanthrol	484-17-3	1222.67	265.00	0.00037±0.00010	0.00141±0.00050↑	0.00051±0.00021↓
48		diflubenzuron	35367-38-5	178.00	309.00	0.05950±0.01270	0.09918±0.01191↑	0.10952±0.04039
49		sulfuric acid	7664-93-9	541.30	281.00	0.04229±0.00592	0.01992±0.00557↓	0.01506±0.00352
50		1-hydroxy-2-naphthoic acid	86-48-6	1142.00	326.00	0.00577±0.00037	0.00042±0.00020↓	0.00434±0.00352
51		aconitic acid	4023-65-8	976.80	83.00	0.00218±0.00050	0.00698±0.00257↑	0.00615±0.00039
52		2-keto-isovaleric acid 1	759-05-7	496.20	141.00	0.00610±0.00064	0.00590±0.00040	0.00700±0.00022↑
53		pterin	2236-60-4	202.20	162.00	0.11990±0.02684	0.23560±0.03798↑	0.13706±0.13160
54		n-acetyl-beta-alanine 2	3025-95-4	734.02	89.00	0.00169±0.00028	0.00130±0.00017	0.00164±0.00009↑
55		pyrimidine	25247-63-6	130.00	81.04	0.00362±0.00077	0.00487±0.00049	0.00377±0.00046↓

56	2,2,4,4,6,6-hexamethyl-1,3,5-trithiane	828-26-2	31.11	223.06	0.00218±0.00100	0.00173±0.00006	0.00236±0.00033↑
57	sucrose	57-50-1	384.24	365.10	0.00071±0.00023	0.00083±0.00009	0.00051±0.00011↓

Note:
Differential metabolites in yellow means they are associated with taste.
Differential metabolites in green means they are associated with flavor.
Blue down arrows indicate the amount decrease at this time point compared with last time point.
Red up arrows indicate the amount increase at this time point compared with last time point.
